# *Xanthomonas campestris* cell–cell signalling molecule DSF (diffusible signal factor) elicits innate immunity in plants and is suppressed by the exopolysaccharide xanthan

**DOI:** 10.1093/jxb/erv377

**Published:** 2015-08-05

**Authors:** Akanksha Kakkar, Narasimha Rao Nizampatnam, Anil Kondreddy, Binod Bihari Pradhan, Subhadeep Chatterjee

**Affiliations:** ^1^Centre for DNA Fingerprinting and Diagnostics, Nampally, Hyderabad 500001, India; ^2^Graduate studies, Manipal University, Manipal, India

**Keywords:** Defence suppressor, elicitor, extracellular polysaccharide, innate immunity, *Nicotiana benthamiana*, quorum sensing, rice, virulence, *Xanthomonas campestris* pv. *campestris*, *Xanthomonas oryzae* pv. *oryzae.*

## Abstract

Several secreted and surface-associated conserved microbial molecules are recognized by the host to mount the defence response. One such evolutionarily well-conserved bacterial process is the production of cell–cell signalling molecules which regulate production of multiple virulence functions by a process known as quorum sensing. Here it is shown that a bacterial fatty acid cell–cell signalling molecule, DSF (diffusible signal factor), elicits innate immunity in plants. The DSF family of signalling molecules are highly conserved among many phytopathogenic bacteria belonging to the genus *Xanthomonas* as well as in opportunistic animal pathogens. Using *Arabidopsis*, *Nicotiana benthamiana*, and rice as model systems, it is shown that DSF induces a hypersensitivity reaction (HR)-like response, programmed cell death, the accumulation of autofluorescent compounds, hydrogen peroxide production, and the expression of the *PATHOGENESIS-RELATED1* (*PR-1*) gene. Furthermore, production of the DSF signalling molecule in *Pseudomonas syringae*, a non-DSF-producing plant pathogen, induces the innate immune response in the *N. benthamiana* host plant and also affects pathogen growth. By pre- and co-inoculation of DSF, it was demonstrated that the DSF-induced plant defence reduces disease severity and pathogen growth in the host plant. In this study, it was further demonstrated that wild-type *Xanthomonas campestris* suppresses the DSF-induced innate immunity by secreting xanthan, the main component of extracellular polysaccharide. The results indicate that plants have evolved to recognize a widely conserved bacterial communication system and may have played a role in the co-evolution of host recognition of the pathogen and the communication machinery.

## Highlight

The *Xanthomonas* conserved cell–cell quorum-sensing molecule elicits innate immunity in plants. The defence response provoked by DSF is suppressed by the exopolysaccharide xanthan, a quorum-sensing-regulated virulence factor.

## Introduction

Plants have evolved the ability to recognize the highly conserved molecular signature characteristic of several secreted and surface-associated microbial molecules and to mount a defence response. These conserved microbial features, known as pathogen-associated molecular patterns (PAMPs) or microbe-associated molecular patterns (MAMPs), act as elicitors; examples are flagellin (Felix *et al.*, 1999), lipopolysaccharide (Newman *et al.*, 2002; Silipo *et al.*, 2005), cold shock protein (Felix and Boller, 2003), and elongation factor Tu (Kunze *et al.*, 2004). The recognition of these elicitors occurs primarily through receptors at the plant cell surface. Recognition of microbial elicitors leads to the innate or basal defence response which restricts pathogen growth in the immediate vicinity of the infected area. However, pathogenic microbes have evolved to suppress or evade the host innate immune response, which is considered to be a pre-condition for their ability to cause disease (for detailed reviews, see Nurnberger *et al.*, 2004; Chisholm *et al.*, 2006; Boller and Felix, 2009). Apart from MAMPs, plants have also developed the ability to recognize endogenous molecules released upon damage caused due to microbial attack, known as damage-associated molecular patterns (DAMPs) (Darvill and Albersheim, 1984; Jha *et al.*, 2007; Aparna *et al.*, 2009).

**Fig. 1.**
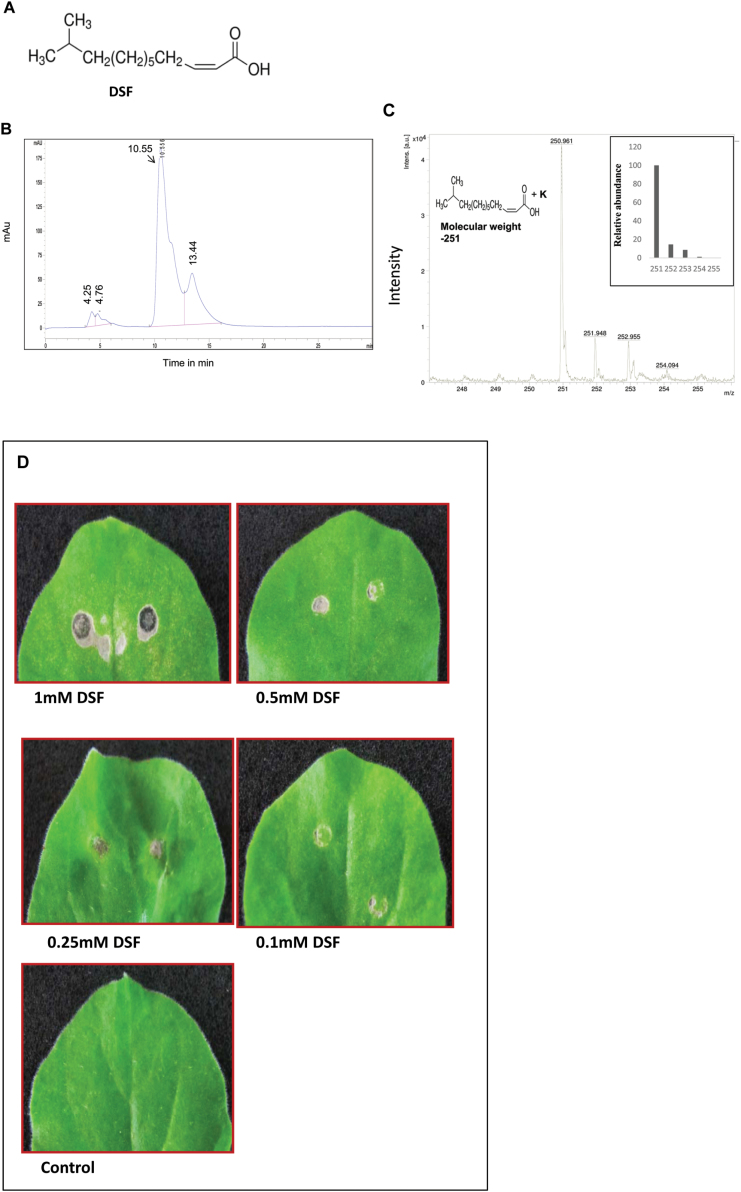
Infiltration of synthetic DSF in *N. benthamiana* induces HR-like symptoms. (A) Chemical structure of DSF, *cis*-​11-​methyl-​2-​dodecenoic acid. (B and C) Analysis of synthetic DSF by (B) HPLC and (C) MALDI–MS. (B) HPLC separation was achieved with an Agilent C18 (4.6 mm×250 mm×5 μm) column. DSF was eluted with water in methanol (20:80, v/v, with 0.1% formic acid) at a flow rate of 0.8ml min^–1^ and was detected at 220nm (retention time 10.55) as described in the Materials and methods. (C) Analysis of the MALDI-MS spectrum was done based on quasimolecular ions. Synthetic DSF gave one quasimolecular ion at 251 *m/z* (inset), which agrees with the calculated mass of a [M+K] ^+^ ion. No fragment ions were present at the applied laser energy. (D) Four-week-old *N*. *benthamiana* leaves were infiltrated with different concentrations of synthetic DSF. Control: 1% methanol in water. Browning of the infiltrated region and HR-like symptoms were observed 24h post-infiltration.

Cell–cell signalling (quorum sensing; QS) is a widespread phenomenon by which bacteria co-ordinate multiple social behaviours via the production and perception of diverse cell–cell communication molecules (Fuqua *et al.*, 2001; Ng and Bassler, 2009). An increasing body of research now suggests that QS plays a central role in pathogenesis of several bacterial pathogens as it synchronizes production and secretion of several virulence factors such as extracellular polysaccharide (EPS), cell-wall-hydrolysing enzymes, and adhesins, which are beneficial to the pathogen at high cell density (Ng and Bassler, 2009). In general, many Gram-negative bacteria mediate QS via the production of acyl homoserine lactone (AHL), typical of the most well characterized process of QS in Gram-negative bacteria (Fuqua *et al.*, 1994, 2001). In contrast, QS in several Gram-negative bacteria belonging to the genera *Xanthomonas* and *Burkholderia* is mediated by the synthesis and perception of a fatty acid signalling molecule (*cis*-11-methyl-2-dodecenoic acid; Fig. 1A) called DSF (diffusible signal factor) (Deng *et al.*, 2011; Ryan and Dow, 2011). It has been shown that in *Xanthomonas* and other closely related bacteria, such as *Xylella fastidiosa* (a pathogen of grapes), synthesis of a DSF family of signalling molecules requires *rpfF* (regulation of pathogenecity factor F), which encodes DSF synthase (RpfF), a bifunctional crotonase having both dehydratase and thioesterase activities (Wang *et al.*, 2004; He *et al.*, 2010; Bi *et al.*, 2012; Beaulieu *et al.*, 2013). RpfF is involved in the synthesis of the DSF family of signalling molecules which are typically *cis*-2-unsaturated fatty acids with a chain length varying from 12 to 14 carbons (Deng *et al.*, 2011).

DSF mediated cell–cell signalling has been reported to play an important role in the virulence of several members belonging to the *Xanthomonas* group of phytopathogens such as *Xanthomonas campestris* pv. *campestris* (*Xcc*; a pathogen of cruciferous plants; Barber *et al.*, 1997), *Xanthomonas oryzae* pv. *oryzae* (*Xoo*; a pathogen of rice; Chatterjee and Sonti, 2002), *Xanthomonas citri* subsp. *citri* (*Xac*; a pathogen of citrus; Siciliano *et al.*, 2006 **),** and *Xanthomonas axonopodis* pv *glycines* (*Xag*; a pathogen of soybean; Thowthampitak *et al.*, 2008). Recent studies indicate that RpfF–DSF mediated cell–cell signalling is widespread in many pathogenic bacteria, including opportunistic human pathogens such as *Stenotrophomonas maltophilia* and *Burkholderia* sp. (Deng *et al.*, 2011; Ryan and Dow, 2011). In addition, DSF family signals have been implicated in interspecies and interkingdom signalling, where they exert inhibitory activity against *Candida albicans* morphological transition, and influence biofilm formation and antibiotic tolerance in *Pseudomonas aeruginosa* (Boon *et al.*, 2008; Deng *et al.*, 2011; Ryan and Dow, 2011).

*Xcc* is an important pathogen belonging to the genus *Xanthomonas* and causes serious disease in cruciferous plants (Mansfield *et al.*, 2012). In *Xcc*, DSF mediated cell–cell signalling regulates production of several secreted virulence factors such as the EPS xanthan, extracellular cell-wall-hydrolysing enzymes, and glucan (Barber *et al.*, 1997). In *Xcc*, xanthan and glucan have been shown to play an important role in the disease process, including suppression of the host innate immune response (Yun *et al.*, 2006; Rigano *et al.*, 2007). It has been proposed that *Xcc* xanthan suppresses the plant defence response, presumably by inhibiting callose deposition (Yun *et al.*, 2006). DSF mediated cell–cell signalling plays a role in the production of an as yet unknown extracellular factor which is involved in suppression of pathogen-induced stomatal closure, a part of the plant innate immune response (Gudesblat *et al.*, 2009).

In this work, it was observed that infiltration of DSF extracts from the cell-free culture supernatant from the wild-type *Xcc*8004 strain induced hypersensitive response (HR)-like symptoms in *Nicotiana benthamiana* leaves (Supplementary Fig. S1A available at *JXB* online). However, extracts from the DSF synthase-deficient mutant of *Xcc* (*rpfF*), which is deficient in the production of DSF, exhibited reduced ability to induce HR-like symptoms (Supplementary Fig. S1B). Interestingly, it was also observed that co-inoculation of *N*. *benthamiana* leaves with DSF and the wild-type *Xcc*8004 strain suppressed the HR-like symptoms induced by the wild-type extract alone (Supplementary Fig. S1D). These results prompted further examination of the role of DSF in *Xanthomonas*–host plant interaction and investigation of whether the DSF signal molecule itself could act as an elicitor of the defence response. Overall, the results clearly indicate that DSF induces plant defence responses such as callose deposition, cell death-associated nuclear fragmentation, and resistance against subsequent infection by pathogenic bacteria. It was also shown that wild-type *Xcc* can suppress the DSF-induced defence response by the production of the EPS xanthan, a DSF-regulated important virulence factor of *X. campestris*.

## Materials and methods

### Bacterial strains and culture conditions

*Xcc* strains were grown on peptone sucrose agar (PSA) or in PS broth at 28 °C shaking at 200rpm, as described previously (Tsuchiya *et al.*, 1982). *Pseudomonas syringae* pv. *syringae* (*Pss*) strain B728a (Loper and Lindow, 1987) was grown in King’s B (KB) medium or KB agar (King *et al.*, 1954) at 28 °C. *Escherichia coli* strains were grown in Luria–Bertani (LB) medium at 37 °C. DSF and AHL used in this study were *cis*-11-methyl-2-dodecenoic acid and 3-oxo-hexanoyl-homoserine lactone (3OC6-HSL; Sigma Aldrich, St. Louis, MO, USA) respectively. AHL and DSF stock solutions were prepared in methanol and stored at –20 °C. Stocks of 3OC6-HSL and DSF were freshly diluted in water and used for plant infiltration and the biosensor assay. The concentrations of antibiotics used were: rifampicin, 50 μg ml^–1^; rifampin, 100 μg ml^–1^; spectinomycin, 20 μg ml^–1^; and tetracycline, 15 μg ml^–1^.

The purity of the synthetic DSF (Sigma Aldrich) was checked using an Agilent 1100 series high-performance liquid chromatography (HPLC) system (Agilent, USA). Data recording and processing was done using chemstation software (Agilent 1100). Separation was achieved with an Agilent C18 (4.6 mm×250 mm×5 μm) column. DSF was eluted with water in methanol (20:80, v/v, with 0.1% formic acid) at a flow rate of 0.8ml min^–1^ and was detected at 220nm (retention time 10.55). The final concentration of the DSF injected into the column was 100 μM. For matrix-assisted laser desorption ionization-mass spectrometry (MALDI-MS) analysis, the synthetic lyophilized DSF sample was redissolved in methanol and then diluted with an appropriate volume of α-cyano-4-hydroxycinnamic acid (HCCA) matrix solution (1:5, v/v). A 1 μl aliquot of the resulting solution was deposited onto a stainless steel target, and the solvent was evaporated under a gentle stream of warm air. The experiment was done on a time-of-flight mass spectrometer (Brukner Daltronics ultraflex III, Germany) equipped with nitrogen laser at 337nm wavelength, 2.5 ns pulse width. After selection of the appropriate site on the target plate by microscopy, the laser light was focused onto the sample–matrix mixture at an angle of 45 ° and a power level of 10^6^–10^7^ W cm^–2^. Positive ions were extracted by a 5–10 keV acceleration potential, focused by a lens, and mass separated in a reﬂectron time-of-ﬂight instrument. At the detector, ions were post-accelerated to 20 keV for maximum detection efficiency. The spectrum was acquired using flex control analysis–flex analysis software. Analysis of the spectrum was done based on quasimolecular ions obtained by the MALDI-MS method. Theoretical mass was calculated with unit mass resolution using an isotope distribution calculator and mass spec plotter by Scientific Instrument Services (SIS; http://www.sisweb.com/mstools/isotope.htm). Analysis for this DSF gave one quasimolecular ion at 251 *m/z*, which agrees with the calculated mass for an [M+K]^+^ ion. No fragment ions were present at the applied laser energy.

### DSF and AHL biosensor assay

For DSF and AHL production assays, *Xcc*8523 (DSF-deficient *rpfF* mutant), harbouring the DSF biosensor plasmid pKLN55 (*Peng: gfp*), and the *E. coli* AHL biosensor strain, JB524 (Wu *et al.*, 2000), harbouring the *Vibrio fischeri luxR* and PluxI:gfpmut3*, were grown in PS and LB media respectively, supplemented with appropriate antibiotics as described previously (Newman *et al.*, 2004, 2008; Shepherd and Lindow, 2009). Cultures of biosensor strains were grown until early log phase, and ethyl acetate samples to be assayed for AHL and DSF were added and incubated overnight at 28 °C with shaking. Green fluorescent protein (GFP) fluorescence intensity (excitation 472nm, emission 512nm) was measured using a Varioskan^®^ Flash fluorescence spectrophotometer (Thermo Fisher Scientific, Vantaa, Finland). Raw fluorescence units were normalized for the optical density at 600nm (OD_600_) values of the biosensor cultures. DSF and AHL concentrations were quantified by comparing normalized fluorescence values with those obtained from standard curves derived from biosensor cells incubated with known concentrations of DSF and AHL standards.

Separation and detection of DSF and AHL on thin-layer chromatography (TLC) plates were done as described previously (Wu *et al.*, 2000; Newman *et al.*, 2008). Ethyl acetate extracts from the culture supernatant of *Xcc* and *Pss*B728a strains were spotted on C-18 reverse-phase silica TLC plates (Merck, Germany), along with 50 μM synthetic DSF and 3OC6-HSL as standards. TLC plates were resolved with 70% methanol. The plates were later dried and overlaid with a thin layer of 0.7% PS and LB agar containing 10^8^ cells ml^–1^ of either the DSF indicator strain 8523 (pKLN55) or the *E. coli* AHL biosensor strain JB524. The plates were then also sprayed with a suspension of the indicator strain as above. Plates were incubated at 28 °C for 48h, and GFP fluorescence was visualized under UV illumination.

### *Nicotiana benthamiana*, *Arabidopsis*, and rice inoculatons

*Nicotiana benthamiana* plants were grown in soil in a growth chamber (Conviron, USA) with 70% humidity, 16h of light and 8h dark, and a temperature of 22 °C. For *N*. *benthamiana*, 4-week old plants were used for the experiments. *Arabidopsis thaliana* ecotype Col-0 were grown on a vermiculate and perlite mixture. For *A*. *thaliana*, 5-week-old plants were used for the experiments. Rice [susceptible cv. Taichung Native-1 (TN-1)] plants were grown in a growth chamber (Conviron) with a temperature setting of 28 °C/25 °C (day/night) and a light intensity of 350 μmol m^–2^ s^–1^. Ten-day-old seedlings were used for callose deposition assay. For infection studies, 30-day-old rice seedlings were used.

Bacterial growth and callose deposition assays for *N*. *benthamiana*, *Arabidopsis*, and rice were done as described previously (Hauck *et al.*, 2003; Yun *et al.*, 2006; Jha *et al.*, 2007; Rigano *et al.*, 2007). All plant inoculations involved a minimum of three replicates. For *N. benthamiana*, leaves of 4-week-old plants were inoculated by syringe infiltration with *Xcc* and *Pss* strains (10^6^ cfu ml^–1^ in water) alone or co-infiltrated with DSF (100 μM) and xanthan (0.5mg ml^–1^). For DSF and xanthan treatment experiments, leaves were infiltrated with water (control), synthetic DSF (50 μM to 1mM; for dose–response experiments), and xanthan (0.5mg ml^–1^). For co-infiltration experiments, 100 μM DSF was infiltrated with xanthan (0.5mg ml^–1^) or with *Xcc* strains 8004, 8523 (DSF-deficient *rpfF* mutant), *gumD* and *gumK* (EPS-deficient transposon-induced mutants of *Xcc*). For xanthan complementation experiments, *gumD* and *gumK* mutants were co-infiltrated with either xanthan alone or xanthan+DSF. To monitor growth of *Pss* strains harbouring either the vector control or the *Xcc* DSF biosynthetic gene *rpfF*, leaves were infiltrated with 1×10^6^ cfu ml^–1^ of bacterial suspension in water with or without exogenous *Xcc* xanthan (0.5mg ml^–1^). Three 1cm^2^ leaf discs from each leaf (three leaves each from two independent experiments) were harvested from the inoculated area at 0, 24, and 48h after inoculation, surface sterilized using sodium hypochlorite (40% solution in water) and 50% ethanol for 1min each, homogenized in 1ml of sterile water using a mortar and pestle, and dilution plated on appropriate antibiotic medium to determine cfu cm^–2^. For *Xcc* co-inoculation experiments, the leaves were infiltrated with *Xcc*8004 (1×10^6^ cfu ml^–1^) with or without 100 μM DSF or xanthan (0.5mg ml^–1^). For the pre-infiltration experiment, leaves were syringe infiltrated with either water (control), 100 μM DSF, or DSF+xanthan, 16h prior to inoculation with a 10^6^ cfu ml^–1^ suspension of the wild-type *Xcc*8004 strain. To monitor *Xcc* growth, leaf discs were harvested as described above to determine cfu cm^–2^. Water soaking-like disease symptoms were monitored at 4 d post-inoculation.

Rice inoculation and resistance assays were done as described previously (Jha *et al.*, 2007). The adaxial surfaces of 10-day-old rice (TN-1) leaves were infiltrated individually with either water or DSF (100 μM) using a 1ml hypodermic syringe without the needle, and examined for callose depositions. For disease resistance assay, the midveins of 30-day-old rice leaves were injected with 50 μl of either DSF (100 μM), cellulase (0.1mg ml^–1^), or buffer (10mM potassium phosphate buffer, pH 6.0). After 24h, the *Xoo* wild-type strain (BXO43) was inoculated onto the midvein, 1–2cm above the point of initial injection, by pricking with a needle that had been used to touch a fresh bacterial colony. After 12 d, the leaves were observed for the appearance of visible disease lesions (discoloration of the midvein and surrounding regions). For *Arabidopsis*, leaves of 5-week-old plants were infiltrated with water (control) or synthetic DSF (50 μM–1mM; for dose–response experiments), or co-infiltrated with 100 μM DSF and xanthan (0.5mg ml^–1^).

### HR assay in *N. benthamiana*

The leaves were infiltrated with either ethyl acetate extracts isolated from the culture supernatants of *Xcc* strains [*Xcc*8004 (wild type), *Xcc*8523 (DSF-deficient *rpfF* mutant)] or synthetic DSF (50 μM to 1mM; for dose–response experiments). For HR suppression experiments, either DSF isolated from the culture supernatants or synthetic DSF (50 μM to 1mM) was co-infiltrated with a 1×10^7^ cfu ml^–1^ suspension of the *Xcc*8004 wild-type strain in water with a needleless hypodermic syringe. HR-like symptoms (browning of the infiltrated area) were observed 24h after infiltration. To detect cell death, H_2_O_2_ accumulation, and autofluorescence, leaves of 4-week-old *N*. b*enthamiana* plants were infiltrated with 100 μM DSF. Leaves were detached at 24h after DSF infiltration and subjected to observation by staining the leaves with trypan blue, by autofluorescence, and by H_2_O_2_ production. Cell death was visualized by lactophenol–trypan blue staining followed by destaining in saturated chloral hydrate as described (Koch and Slusarenko, 1990). Autofluorescence was visualized under a stereo fluorescence microscope (Zeiss Lunar V12; Carl Zeiss, Goettingen, Germany), using an eGFP filter set (excitation 470/40nm, emission 525/50nm). H_2_O_2_ was detected by an *in situ* histochemical staining procedure using 3,3′-diaminobenzidine (DAB) as described previously (Thordal-Christensen *et al.*, 1997). Briefly, the leaves were detached and placed in a solution containing DAB (1mg ml^–1^; pH 5.5) for 2h at room temperature. The leaves were boiled in 95% ethanol for 2min and stored in distilled water. H_2_O_2_ production was visualized as a reddish-brown coloration.

### Callose staining

Bacteria or synthetic DSF were infiltrated into *N. benthamiana*, *Arabidopsis*, and rice leaves as described above. Callose staining was performed at 18h after inoculation as described by Hauck *et al.* (2003). After 24h, the leaves were cleared of chlorophyll using alcoholic lactophenol, rinsed in 50% ethanol and then in water, before staining for 2h with 0.02% aniline blue (Sigma Aldrich) in 150mM K_2_HPO_4_, pH 9.5. Samples were mounted in 50% glycerol and were visualized by a stereo fluorescence microscope (Zeiss Lunar V12; Carl Zeiss), using a blue filter (excitation wavelength 365nm and emission wavelength >420nm), long band pass (BP), and ×20 objective. The number of callose deposits per 0.5mm^2^ area surrounding the infiltrated zone were counted using Axio Vision Rel 4.8 image analysis software (Carl Zeiss).

### Rice and *Arabidopsis* root cell death assays

The root cell death assay was carried out as described previously (Jha *et al.*, 2007; Aparna *et al.*, 2009). *Arabidopsis* and rice root tips, 1–2cm long, were treated with water, 100 μM DSF, and cellulase (0.1mg ml^–1^). After incubation for 3–4h, roots were washed in 1× phosphate-buffered saline (PBS) and stained with propidium iodide (PI) by vacuum infiltration for 10–15min. The roots were mounted on a microscope slide in 50% glycerol in 1× PBS; 0.3 μm thick longitudinal optical sections were acquired on a Zeiss LSM-510 Meta confocal microscope using a plan ApoChromat ×63/1.4 oil objective and were further projected to obtain the image of 2–3 μm total thickness. An HeNe laser at 543nm excitation and emission >560nm (LP) was used to detect PI internalization. The images were analysed using the Zeiss LSM image examiner software.

### Transposon mutagenesis and screening for mutants altered in suppression of DSF-induced HR-like symptoms

Transposon (Tn*5*) mutagenesis was done in the *Xcc*8004 wild-type background by introducing the transposon mutagenesis suicidal plasmid pRL27 from *E. coli* by conjugation as described before (Larsen *et al.*, 2002). Transposon mutants were selected on PSA containing 50 μg ml^–1^ kanamycin. A total of 3000 colonies from 10 independent matings were screened by co-infiltration with 0.5mM DSF on leaves of 4-week-old *N*. *benthamiana* plants with a needleless syringe. HR-like symptoms were scored manually 24h post-inoculation by comparing the leaves treated with DSF alone or co-infiltrated with the wild-type *Xcc*8004 strain.

Two colonies (SC130 and SC235) were identified that were unable to suppress DSF-induced HR-like symptoms, described in detail in the Supplementary Materials and methods at *JXB* online. In general, the rest of the 2998 transposon-induced mutants suppressed the DSF-induced HR response and the majority of them exhibited disease symptoms. The sequence of chromosomal DNA flanking the Tn*5* insertion site was determined as described previously (Larsen *et al.*, 2002). Briefly, genomic DNA from the mutants was digested with *Bam*HI, circularized, and electroporated into *E. coli* DH5α λ*pir* cells. Plasmids recovered from DH5α λ*pir* Km^r^ colonies were sequenced with primers oriented outward from the transposon flanks (Larsen *et al.*, 2002). Sequences obtained from two independent clones were confirmed and analysed by NCBI BLAST X search (Altschul *et al.*, 1997). The transposon insertions in SC130 and SC235 were located at positions corresponding to codon 306 and 83 of *gumD* (Locus tag: XC_1600; 484 a. a) and *gumK* (Locus tag: XC_1667; 400 a. a) respectively. *gumB* and *gumK* are members of the *gum* operon of *Xanthomonas* involved in biosynthesis of the EPS xanthan. To confirm that the phenotype is due to transposon insertion, marker exchange mutagenesis was carried out in the *Xcc*8004 wild-type background. EPS was isolated and quatitated from the wild type, and transposon-induced and marker exchange mutants by the phenol sulphuric acid method as described previously (Rai *et al.*, 2012). Analysis of EPS in the *gumD* and *gumK* mutants indicated that as expected, they were deficient in the production of secreted xanthan.To obtain the *gumD* and Δ*rpfF*-*gumD* double mutant, a 500bp internal fragment of the *gumD* gene was PCR amplified with primers-Scc15 Pk18 Gum F EcoRI, GCGAATTCGTTGTATTCGGTGATCTGCTTC; and Scc16 PK18 GUM R HindIII, GCAAGCTTGCTCGC CAAGCGGCAACGAGATCCAC, and cloned in the pK18mob plasmid (Schafer *et al.*, 1994). The resulting recombinant plasmid was electroporated individually into competent cells of *Xcc*8004 wild type and Δ*rpfF* (Pradhan and Chatterjee, 2014; DSF-deficient deletion mutant) background strains to obtain a Km^r^ single recombinant which was further confirmed by PCR and sequencing. Integration of pK18mob results in non-polar mutation (Windgassen *et al.*, 2000), as the transcriptional orientations of the *lacZ* promoter of the pK18mob and the *gumD* gene fragments were in the same direction so that the mutation caused by the pK18mob plasmid is unlikely to cause any polar effect on the downstream gene. For complementation of the EPS-deficient *gumD* mutant, the wild-type *gumD* gene was amplified from the wild-type *Xcc*8004 strain using primer pairs Scc17 PHM1 F HindIII, GCAAGCTTAGGAGGACAGCT ATGCTTTTGGCAGACTTGAGTAG; and Scc18 PHM1 R Eco RI, GCGAATTCTCAGTACGCGGTCTTCTGTCCGAGC, and cloned in the broad host range pHM1 vector.

### Detection of DSF produced by *Xcc in planta*

To detect *in planta* production of DSF by *Xcc*, *N*. *benthamiana* leaves were infiltrated with either the wild-type *Xcc*8004 (pKLN55) or the 8523 (pKLN55) biosensor strain grown in PS medium overnight (12h) at a density of 1×10^6^ cfu ml^–1^, similar to the inoculation experiments for detecting callose deposition and *in planta* growth. The wild-type *Xcc*8004 (pKLN55) exhibited very low GFP fluorescence, indicative of low DSF levels or production at a density of 1×10^6^ cfu ml^–1^ (Pradhan and Chatterjee, 2014). For estimating DSF levels *in planta*, the *rpfF* mutant strain 8523 (pKLN55) (GFP^–^) was co-infiltrated with different concentrations of DSF (in the range of 10–100 μM). Infiltrated leaves of *N*. *benthamiana* were analysed by confocal laser scanning microscopy (CLSM; LSM 510, meta; Carl Zeiss) at 48h post-infiltration using a plan ApoChromat ×100 oil objective. The excitation maximum was at 488nm (argon laser) and the emission maxima were observed in BP 510–530nm (for eGFP fluorescence) and BP 650–710nm (for red autofluorescence of leaf). The mean GFP fluorescence intensity of ~50 bacterial cells of 8523 (pKLN55) was measured and compared with the mean GFP fluorescence intensity of wild-type *Xcc*8004 (pKLN55) in *N*. *benthamiana* leaves at 0, 1, 2, 3, and 4 d post-inoculation.

### Pre-infiltration with DSF and subsequent challenge with flg22

Leaves of 4-week-old *N*. *benthamiana* plants were pre-infiltrated with 10 μM DSF or 1% methanol (solvent control) for 16h prior to challenge with 100nM flg22 (Genescript, Corp, USA, Cat. no. RP19986) for 18h and stained with aniline blue to visualize callose deposition by epifluorescence microscopy.

### RNA gel blot analysis

Total RNA was isolated from *N. benthamiana* leaves using Trizol (Invitrogen, CA, USA). A 20 μg aliquot of total RNA from each sample was separated on a 1.2% agarose gel containing formaldehyde, and transferred to a Hybond-N^+^ nylon membrane. The filters were pre-hybridized and hybridized in hybridization buffer: 7% (w/v) SDS, 0.5M phosphate buffer, pH 7.2. Pre-hybridization was performed at 65 °C for 2h. Hybridization was performed overnight with the addition of a denatured ^32^P-labelled Nb *PR-1* probe that was synthesized using the random primer labelling kit (BRIT, Jonaki, India). Membrane washing was performed according to standard protocols. The filters were exposed to storage phosphor screen autoradiography and screened in a BAS 2500 Fuji phosphor imaging system.

## Results

### The cell–cell signalling molecule diffusible signal factor (DSF) from *Xanthomonas induces* plant defence response

To understand the effect of DSF on the host plant, synthetic DSF, *cis*-​11-​methyl-​2-​dodecenoic acid (see the Materials and methods), was obtained and examined by HPLC and MALDI-MS analysis (Fig. 1A–C). HPLC and MALDI-MS analysis confirmed that the synthetic DSF was pure. The bioactivity of synthetic DSF was also examined using an *Xcc* DSF biosensor strain *Xcc*8523 (pKLN55), a DSF-deficient mutant harbouring the *gfp* reporter responsive to exogenous DSF (Newman *et al.*, 2004). The addition of synthetic DSF could complement the expression of the DSF-responsive GFP reporter in a dose-dependent manner (Supplementary Fig. S2 at *JXB* online).

**Fig. 2.**
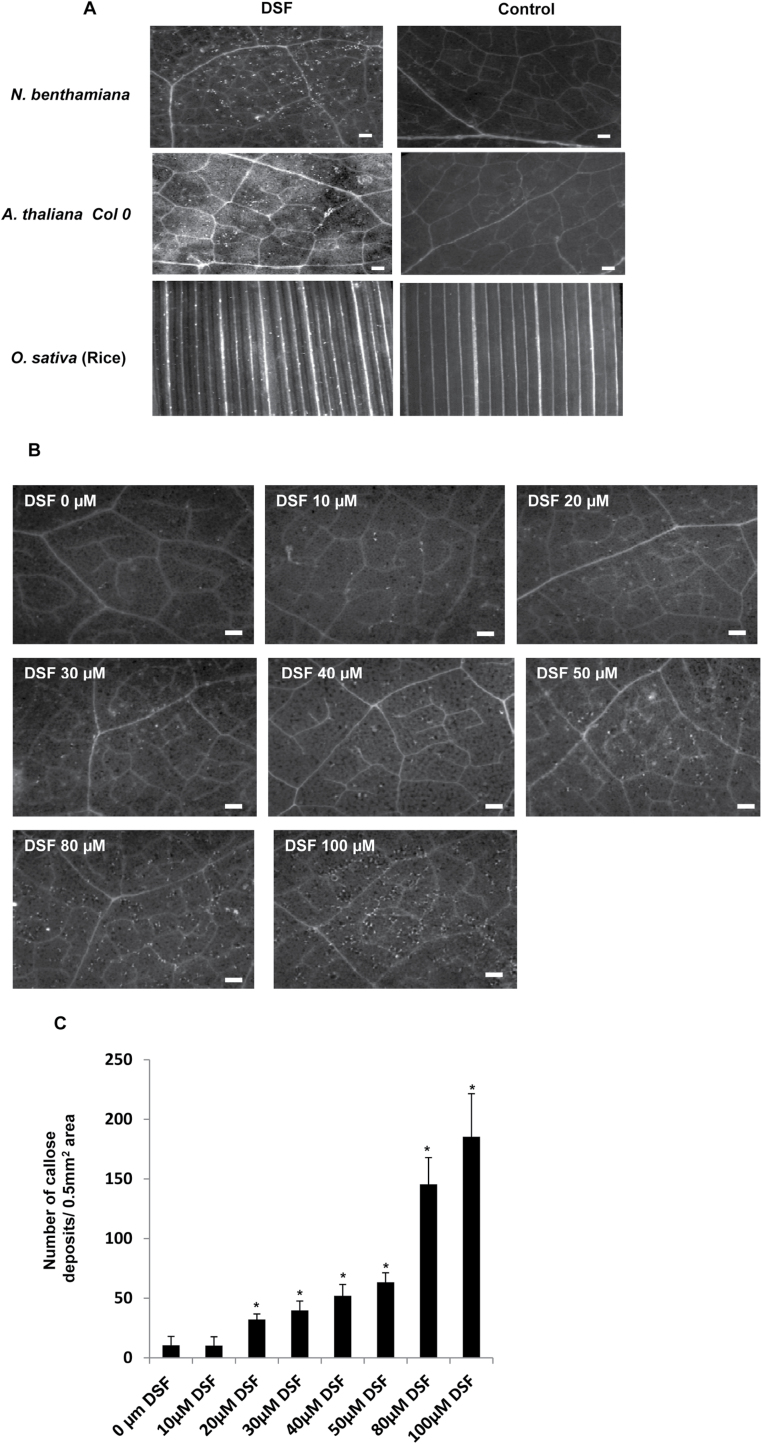
DSF induces callose deposition. (A) Infiltration of 100 μM synthetic DSF (*cis*-11-methyl-2-dodecenoic acid) induces callose deposition in *N*. *benthamiana*, *Arabidopsis thaliana* (Col-0), and rice (*Oryzae sativa*). Callose deposition was visualized 18h post-infiltration by staining the leaves with aniline blue and examination using an epifluorescence microscope. White dots in these pictures are indicative of callose deposition. (B) DSF induced callose deposition in *N. benthamiana* leaves in a dose-dependent manner. *Nicotiana benthamiana* leaves were infiltrated with (left to right) 10, 20, 30, 40, 50, 80, and 100 μM DSF, and control (0 μM; 1% methanol in water), and visualized for callose deposition 18h post-infiltration. Scale bars=500 μm. (C) Average number of callose deposits per 0.5mm^2^. Error bars represent SD values from four leaves of each plant in three independent experiments. Six microscopic fields from each leaf were analysed. *P<0.01, significant differences between the responses to the DSF treatment compared with the control (indicated by 0 μM) as assessed by Student’s *t*-test.

Infiltration of *N*. *benthamiana* leaves with synthetic DSF induced browning of the infiltrated region, which appeared similar to HR-like symptoms, in a dose-dependent manner (Fig. 1D). Visible HR-like symptoms (browning) were observed with a concentration of DSF in the range of 0.25–1mM, 24h post-infiltration. Callose deposition has been used as a marker for plant basal defence response and has been reported to play a role in the plant defence in limiting pathogen growth (Bestwick *et al.*, 1995; Adam and Somerville, 1996; Hann and Rathjen, 2007). In order to examine the ability of DSF to induce callose deposition in plants, synthetic DSF was infiltrated in *N*. *benthamiana*, *Arabidopsis*, and rice leaves, at a concentration of 10–200 μM. Callose staining 18h post-inoculation indicated that DSF induced callose deposition in a dose-dependent manner in *N*. *benthamiana*, *Arabidopsis*, and rice (Fig. 2; Supplementary Fig. S3; Supplementary Table S1 at *JXB* online). It was observed that the *N*. *benthamiana* leaves infiltrated with a DSF concentration of ≥20 μM exhibited significantly higher callose deposition compared with those infiltrated with either control (1% methanol in water) or a DSF concentration <10 μM (Fig. 2). This effect was specific to DSF as infiltration of a range of compounds with a related structure, including *trans*-11-methyl dodecenoic acid, decanoic acid, lauric acid, palmitic acid, myristoleic acid, and palmitoleic acid, on *N*. *benthamiana* leaves resulted in very little or no callose deposition (Supplementary Table S2).

In a recent study, it was demonstrated that plants pre-treated with a lower concentration of AHL (QS signalling molecule) exhibited an induced defence response (MTI; MAMP-triggered immunity) on subsequent challenge with flg22 (Schenk *et al.*, 2014). To see whether DSF can influence MTI, the callose deposition was examined in plants that were pre-infiltrated with 10 μM DSF or 1% methanol (solvent control) for 16h prior to challenge with 100nM flg22. Interestingly, leaves pre-infiltrated with DSF showed a substantially higher amount of callose deposition compared with leaves pre-infiltrated with solvent control and subsequently challenged with flg22 (Supplementary Fig. S4 at *JXB* online). It is important to note in this regard that infiltration of 10 μM DSF alone did not induce a significant amount of callose deposition compared with the solvent control (Fig. 2; Supplementary Fig. S4). This suggests that the application of a lower concentration of DSF may prime plants and influence their subsequent defence response.

**Fig. 3.**
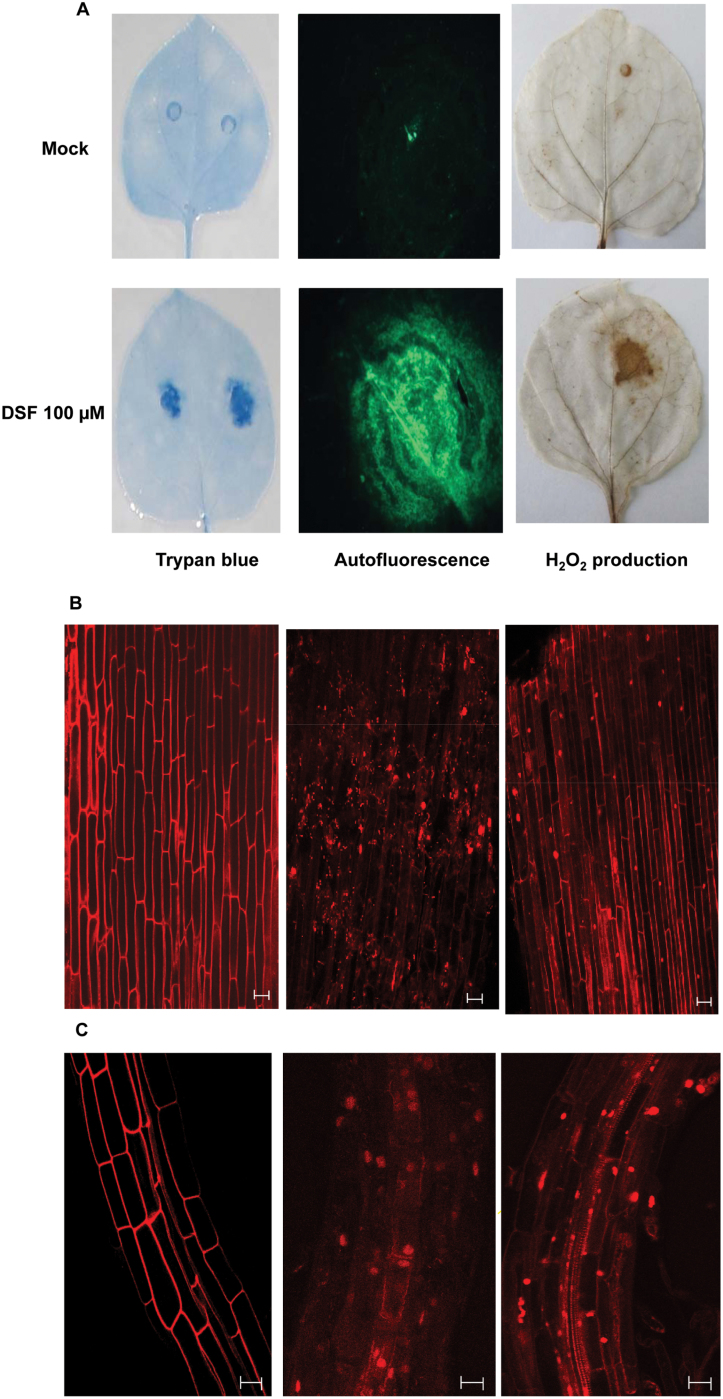
DSF induced cell death in *N*. *benthamiana*, rice, and *Arabidopsis*. (A) DSF induced cell death in *N*. *benthamiana*. Leaves of 4-week old plants were infiltrated with 100 μM DSF. The leaves were detached at 24h after DSF infiltration and subjected to observation by staining the leaves with trypan blue, an indicator of cell death (left); autofluorescence (centre); and H_2_O_2_ production (right). H_2_O_2_ accumulation was visualized by staining with diaminobenzidine (DAB), a histochemical reagent for *in situ* detection of H_2_O_2._ Six or more leaves were examined for each condition, and representative fields are shown. (B and C) DSF induced cell death in rice and *Arabidopsis* roots. Isolated roots were treated with DSF, cellulase, and control (buffer-treated) for 16h, stained with propidium iodide (PI), and examined under a confocal microscope. Cellulase (a potential DAMP) from *Aspergilus niger* was used as a positive control. (B) From left to right, rice root tips, 1–2cm long, from 3- to 4-day-old seedlings were treated with control (methanol in water), 100 μM DSF, and cellulase (0.2mg ml^–1^) from *A. niger*. (C) From left to right, *Arabidopsis* roots treated with water control, DSF (100 μM), and cellulase. Treatment with water control did not induce cell death (intake of PI), whereas treatment with either DSF or cellulase induced cell death. Similar results were obtained in at least three independent experiments. Scale bars=20 μm.

Earlier reports indicated that localized programmed cell death (PCD) reactions are associated with the plant defence response (Pennell and Lamb, 1997). Plant cells undergoing PCD such as the HR-like response exhibit autofluorescence due to deposition of lignin-like compounds and H_2_O_2_ accumulation (Stone *et al.*, 2000). Cell death, autofluorescence, and H_2_O_2_ accumulation were thus examined in *N*. *benthamiana* leaves infiltrated with 100 μM DSF. Examination of leaves infiltrated with DSF indicated a significant increase in trypan blue staining, autofluoresence, and H_2_O_2_ accumulation (visualized by DAB), whereas the control treatments did not exhibit these effects (Fig. 3A). It has been demonstrated that rice root cells exhibit PCD upon exposure to cell-wall-hydrolysing enzymes (such as cellulase and lipase), which are potential DAMPs (Jha *et al.*, 2007; Aparna *et al.*, 2009). To assess the ability of DSF to induce cell death, rice and *Arabidopsis* roots were treated with DSF, stained with PI, and examined by CLSM. Internalization of PI is indicative of cell death and it is excluded by live cells. The control buffer-treated roots exhibited a prominent cell wall-associated autofluorescence but no internalization of PI into the cells (Fig. 3B). Treatment of rice and *Arabidopsis* roots with 100 μM DSF resulted in intake of PI, indicating compromised integrity of the plasma membrane (Fig. 3B).

### Production of DSF is associated with induced defence response

**Fig. 4.**
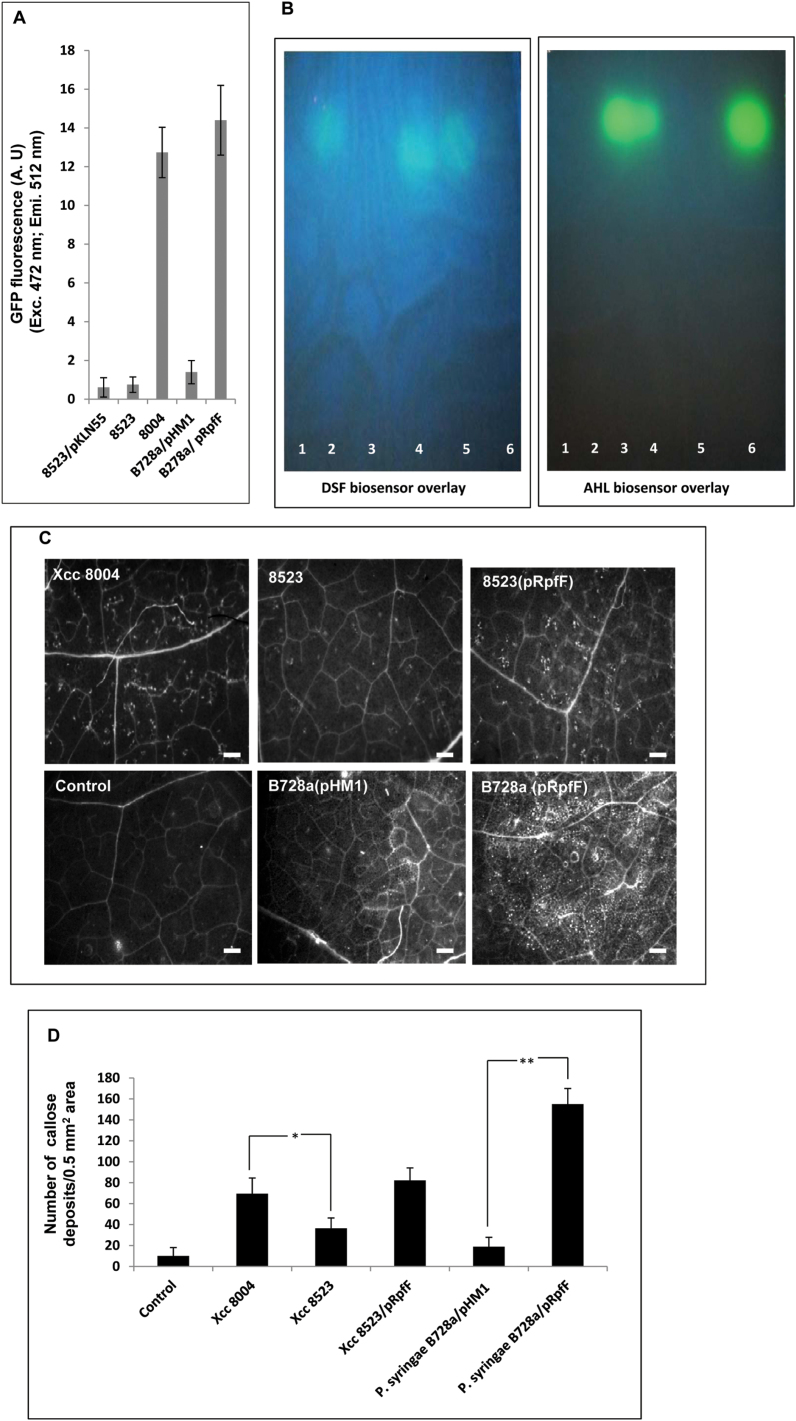
Production of DSF is associated with induced callose deposition. (A) Assay for DSF production by different bacterial strains using the *Xcc* DSF biosensor 8523 (pKLN55) in which the DSF-responsive promoter from the endoglucanase gene is cloned upstream of an eGFP reporter (P_*eng*_:GFP). DSF extracted from the cell-free culture supernatant of *Xcc*8004 (wild type, DSF^+^); 8523 (DSF^–^); *Pseudomonas syringae* pv. *syringae Pss*B728a (pHM1) (wild-type strain harbouring the empty vector); and *Pss*B728a (pRpfF) (*Pss* harbouring the *Xcc* DSF synthetic gene *rpfF*). An increase in GFP fluorescence (represented on the *y*-axis) compared with the control (extracts from the *Xcc*8523; *rpfF* mutant) indicates the amount of DSF produced by different strains. (B) TLC analysis for the production of DSF and AHL in *Xcc* and *Pss*B728a. TLC was performed with crude ethyl acetate extracts isolated from the culture supernatant of different strains of *Xcc* and *Pss*. DSF (left panel) and AHL (right panel) were detected by an overlay of the *Xcc* DSF indicator strain 8523 (pKLN55) and the *E. coli* AHL biosensor (JB524) on TLC plates. The photograph was taken with the UV light source oriented from the top of the TLC plates. Column 1, *Xcc*8523 (DSF-deficient mutant); column 2, *Xcc*8004 (*Xcc* wild-type strain); column 3, *Pss*B728a (pHM1); column 4, *P*ssB728a (pRpfF); column 5, synthetic DSF (20 μM *cis*-11-methyl-2-dodecenoic acid); column 6, synthetic AHL (10 μM *N*-3-oxo-hexanoyl-dl-homoserine lactone; 3OC6-HSL). Similar results were obtained in three independent experiments. (C) From left to right, callose deposition in *N*. *bethamiana* leaves infiltrated with a 1×10^6^ cfu ml^–1^ suspension of different bacterial strains: wild-type *Xcc*8004, 8523, 8523 (pRpfF), water control, *Pss*B728a (pHM1), and B728a (pRpfF). Callose deposition was visualized by staining with aniline blue and examined using a stereo fluorescence microscope 24h post-inoculation. White dots in these pictures are indicative of callose deposition. (D) Average number of callose deposits per 0.5mm^2^ area. Error bars represent SD values from three leaves of each plant from three independent experiments. Six microscopic fields from each leaf were analysed. Differences between the responses to *Xcc*8523 (DSF^–^ mutant; indicated by *P<0.01) and the *P. syringae* B728a wild-type strain harbouring the *Xcc* DSF synthase *rpfF* (indicated by **P<0.001) compared with the wild-type strains of *Xcc*8004 and *P*. *syringae* B728a were significant as assessed by Student’s *t*-test.

It has been shown previously that callose deposition is required for disease resistance against many pathogens, including *Xcc*, and enhanced callose deposition is associated with increased resistance response in *N*. *benthamiana* to *Xcc* infection (Bestwick *et al.*, 1995; Hauck *et al.*, 2003; Yun *et al.*, 2006; Rigano *et al.*, 2007). In *Xcc*, *rpfF* encodes the DSF synthase (RpfF) which is required for the production of DSF (Fig. 4A, B). In order to investigate whether callose deposition is associated with the production of the cell–cell signalling molecule DSF in *Xcc*, *N*. *benthamiana* leaves were inoculated with a 1×10^6^ cfu ml^–1^ suspension of wild-type *Xcc*8004, *Xcc*8523 (DSF^–^ mutant defective in RpfF), and *Xcc*8523 (pRpfF) (DSF^–^ mutant harbouring the plasmid-borne wild-type *rpfF* allele). The number of callose deposits that are elicited by these strains was quantitated following aniline blue staining and epifluorescence microsopy at 24h post-inoculation (Fig. 4C). Higher amounts of callose deposition (~2-fold) were detected in *N*. *benthamiana* leaves that were infiltrated with wild-type *Xcc*8004 compared with *Xcc*8523 (Fig. 4D). Complementation of *Xcc*8523 with the wild-type *rpfF* gene restored callose deposition to the levels that were induced by the wild-type *Xcc*8004 strain (Fig. 4C, D). To detect DSF levels produced by the wild-type *Xcc* strain in *N*. *benthamiana* leaves, the wild type *Xcc*8004 (pKLN55) DSF biosensor strain was infiltrated under similar conditions at a density of 1×10^6^ cfu ml^–1^. At a low cell density (1×10^6^ cfu ml^–1^), the *Xcc* DSF biosensor strain exhibited low GFP fluorescence (uninduced) in PS medium (Pradhan and Chatterjee, 2014). Analysis of *N*. *benthamiana* leaves by confocal microscopy indicated that the wild-type *Xcc* produced a significant amount of DSF *in planta*, as indicated by the induced DSF-responsive GFP fluorescence at 24–48h post-infiltration (Supplementary Fig. S5 at *JXB* online).

It has been shown previously that synthesis of DSF in *Xanthomonas* and other closely related bacteria requires *rpfF*, which encodes DSF synthase (RpfF), a bifunctional crotonase having both dehydratase and thioesterase activities (Wang *et al.*, 2004; He *et al.*, 2010; Bi *et al.*, 2012; Beaulieu *et al.*, 2013). It has also been demonstrated that recombinant *E. coli* and *Erwinia herbicola* expressing RpfF were able to produce the secreted DSF family of signalling molecules (Bi *et al.*, 2012; Beaulieu *et al.*, 2013). It was thus investigated whether the production of DSF in a non-DSF-producing phytopathogen could provoke a defence response-associated callose deposition and reduce its growth in the host plant. The DSF biosynthetic gene *rpfF* of *Xcc* was expressed under the control of the P_lac_ promoter in *Pss*B728a, a non-DSF-producing pathogen of snap bean and the model plant *N. benthamiana* (Loper and Lindow, 1987; Vinatzer *et al.*, 2006). It has been shown that *Pss* produces the QS signalling AHL, 3OC6-HSL (Quiñones *et al.*, 2004) (Fig. 4A, B). A DSF and AHL biosensor assay, and C18 reverse phase TLC analysis with the ethyl acetate extract from the culture supernatants of the wild-type *Pss*B728a strain harbouring either the *Xcc rpfF* [B728a (pRpfF)] or the vector control [*Pss*B728a (pHM1)], was performed. As expected, the wild-type *Pss*B728a strain harbouring the vector control [*Pss*B728a (pHM1)] produced 3OC6-HSL and did not produce any detectable DSF, while *Pss*B728a (pRpfF) produced 3OC6-HSL as well as DSF (Fig. 4A, B). The migration pattern of DSF extracted from either the wild-type *Xcc*8004 or *Pss*B728a (pRpfF) were similar to that of synthetic DSF (Fig. 4B).

**Fig. 5.**
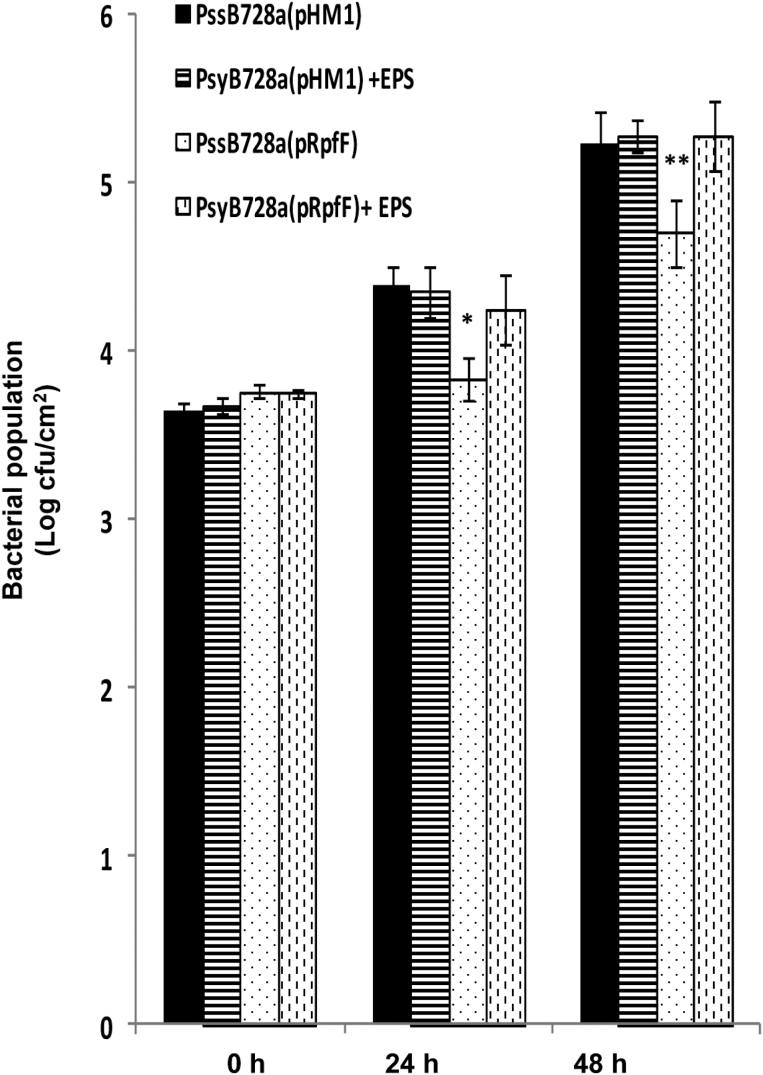
Production of DSF in *Pseudomonas syringae* pv. *syringae* (*Pss*) reduces growth in *N*. *benthamiana* leaves. Leaves of 4-week-old *N. benthamiana* were infiltrated with wild-type *Ps*s harbouring the plasmid containing the DSF synthase (pRpfF) or the empty vector (pHM1) alone or co-inoculated with *Xcc* EPS (xanthan; 0.5mg ml^–1^). The bacterial population was measured at 0, 24, and 48h post-inoculation from six 1cm^2^ leaf disc areas around the infiltration zone. Values presented are the average log (cfu cm^–1^) from six leaves (two independent experiments). A significantly different population of bacteria compared with the wild-type *Pss* harbouring the empty vector control (pHM1) based on a pairwise Student’s *t*-test is indicated with either one or two asterisks: *P≤0.05; **P≤0.02.

Next, *N*. *benthamiana* leaves were infiltrated with the *Pss*B728a wild-type strain harbouring either the *Xcc rpfF* [*Pss*B728a (pRpfF)] or the vector control [*Pss*B728a (pHM1)]. Significantly higher amounts of callose (~6-fold) deposition were detected in B728a (pRpfF) compared with the vector control (Fig. 4C, D). Next, experiments were conducted to determine whether defence responses induced by DSF-producing *Pss*B728a could have an effect on bacterial growth. A bacterial growth assay was carried out in *N*. *benthamiana* leaves infiltrated with a 1×10^6^ cfu ml^–1^ suspension of *Pss*B728a (pRpfF) or vector control. The *in planta* growth assay indicated that wild-type *Pss*B728a harbouring the *Xcc rpfF* [*Pss*B728a (pRpfF)] exhibited reduced growth at 24h and 48h after inoculation compared with the vector control, which although smaller (~5- to 6-fold), was still statistically significant (Fig. 5). However, there was not much difference in growth at 4 d post-inoculation (Supplementary Fig. S6 at *JXB* online).

### The extracellular polysaccharide xanthan suppresses the DSF-induced defence response in *N*. *benthamiana*

**Fig. 6.**
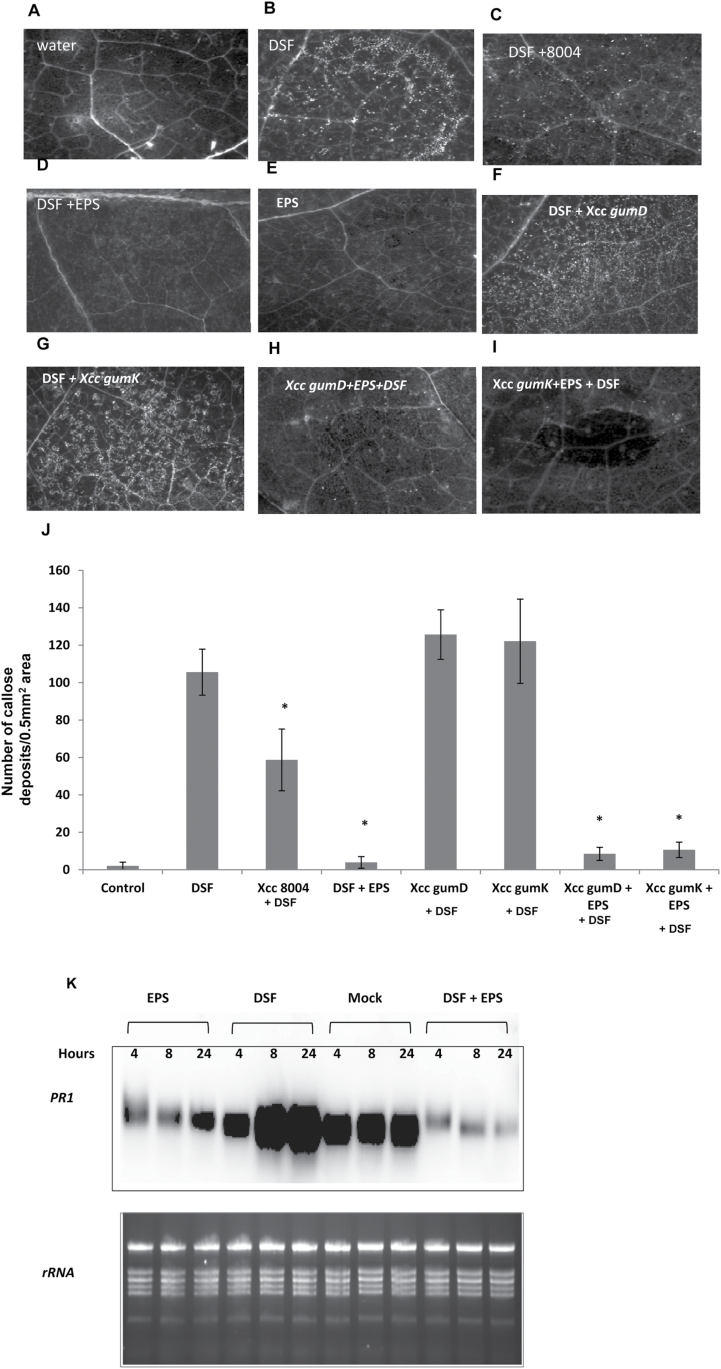
DSF-induced callose deposition was suppressed by either the wild-type *Xcc*8004 or the EPS xanthan. (A–I) Callose deposition in *N*. *benthamiana* leaves after inoculation with water control (A), 100 μM DSF (B), and EPS (E; xanthan; 0.5mg ml^–1^) alone or co-inoculation of DSF with either *Xcc*8004 (C), EPS (D; xanthan), *Xcc gumD* (F), *Xcc* *gumK* (G), *Xcc gumD*+EPS (H), or *Xcc gumK*+EPS (I). Callose deposition was visualized by staining with aniline blue and examined using a stereo fluorescence microscope 24h post-inoculation. For co-inoculation experiments with *Xcc* strains, a bacterial suspension of 1×10^6^ cfu ml^–1^ was used. (J) Average number of callose deposits per 0.5mm^2^ area of *N*. *benthamiana* leaves inoculated with control (water) and DSF alone, or co-inoculation of DSF with either *Xcc*8004, EPS, *Xcc gumD* amd *gumK* (*Xcc* mutants deficient in EPS production), or *Xcc gumD* and *gumK* mutants supplemented with EPS (*gumD* and *gumK*+ EPS). Error bars represent SD values from three leaves of each plant and three independent experiments. Six microscopic fields from each leaf were analysed. * indicates (*P*<0.001) significantly lower callose deposits compared with leaves treated with DSF alone as determined by two-tailed Student’s *t*-test. (K) RNA gel blot analysis of *PR-1* gene expression in *N. benthamiana* leaves infiltrated with water (mock), DSF (100 μM), EPS (xanthan, 0.5mg ml^–1^) alone or co-infiltration with DSF+EPS. RNA was isolated at the indicated times for RNA gel blot analysis. Data shown are representative of those obtained from three independent experiments.

Since it was observed that co-inoculation of the *Xcc* wild-type strain with DSF suppressed DSF-induced HR-like symptoms in *N*. *benthamiana* (Supplementary Fig. S1D at *JXB* online), further experiments were performed to determine whether co-inoculation of the wild-type *Xcc* strain with DSF could also suppress DSF-induced callose deposition. Co-infiltration of *N*. *benthamiana* leaves with wild-type *Xcc*8004+DSF resulted in suppression of callose deposition (Fig. 6B, C). Significantly lower amounts of callose deposition were detected in *N*. *benthamiana* leaves that were co-infiltrated with *Xcc*8004+DSF compared with those infiltrated with DSF alone (Fig. 6J).

To gain more insight into DSF–plant interaction and to identify potential suppressor(s) of the DSF-induced defence response, a transposon-induced mutant library of *Xcc*8004 was screened to isolate mutants altered in their capacity to suppress DSF-induced callose deposition. In this screen, *N*. *benthamiana* leaves were co-inoculated with ~3000 transposon-induced mutants from the library (see the Supplementary Materials and methods at *JXB* online for details) along with DSF, and compared with those infiltrated with DSF alone. Two transposon-induced mutants, *gumD* (*gumD*::*m*Tn*5*) and *gumK* (*gumK*::*m*Tn*5*), that were deficient in their capacity to suppress the DSF-induced callose deposition, were identified (Fig. 6F, G). In *Xanthomonas*, *gumD* and *gumK* are members of the *gum* operon which consists of 12 genes (*gumB*–*gumM*) involved in the synthesis of the EPS xanthan and is highly conserved among *Xanthomonas* species (Büttner and Bonas, 2010). As expected, the transposon-induced mutants (*gumD* and *gumK*) and an insertional mutant *gumD*::pK18mob exhibited reduced production of extracellular xanthan (Supplementary Fig. S7 at *JXB* online). Co-infiltration of *N*. *benthamiana* leaves with purified commercial xanthan (0.5mg ml^–1^; Sigma Aldrich: G1235) together with DSF suppressed the DSF-induced callose deposition (Fig. 6D). Interestingly, co-infiltration of xanthan+*gumD* or *gumK* mutants suppressed the DSF-induced callose deposition (Fig. 6H–J). Previously it was reported that *Xcc* in *N*. *benthamiana* produces copious amount of the EPS xanthan (14–19mg g^–1^ as dry weight) at 2–4 d post-infection (Aslam *et al.*, 2008), which agrees with the concentrations of xanthan used for suppression of the DSF-induced defence response.

It has been shown that induced expression of the gene encoding PR-1 in *N*. *benthamiana* is associated with the resistance response against *Xcc* infection (Rigano *et al.*, 2007). To see whether DSF has any effect on *PR-1* expression, RNA gel blot analysis was performed with RNA extracted from the *N*. *benthamiana* leaves at 4, 8, and 24h after infiltration with either DSF, water (control), xanthan, or xanthan+DSF. In response to control (water), accumulation of *PR-1* transcript did not change much during 4–24h after infiltration (Fig. 6K). In contrast, accumulation of *PR-1* transcript increased to at least 2-fold more after infiltration with DSF at 8–24h, compared with the response to control treatment. Interestingly, infiltration with xanthan alone or co-infiltration of xanthan+DSF produced a significant reduction in *PR-I* expression at 4, 8, and 24h (Fig. 6K). This result suggests that DSF induces expression of *PR-1* in *N*. *benthamiana* while xanthan suppresses *PR-1* expression.

**Fig. 7.**
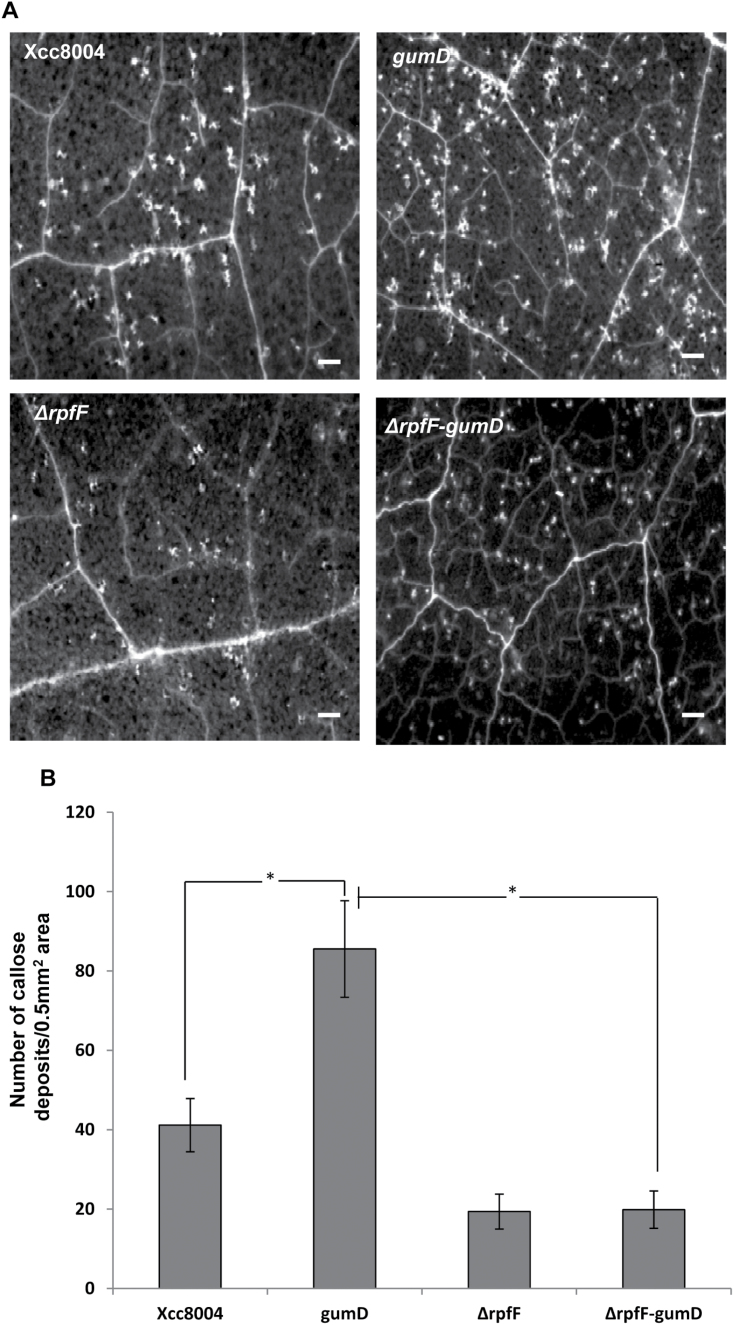
Callose deposition induced by different strains of *Xcc*. (A) *N*. *benthamiana* leaves were infiltrated with a 1×10^6^ cfu ml^–1^ suspension of different *Xcc* strains; *Xcc*8004 (wild type), *gumD* (xanthan-deficient mutant), Δ*rpfF* (DSF-deficient *rpfF* deletion mutant), and the Δ*rpfF-gumD* double mutant. Callose deposition was visualized by staining with aniline blue and examined using a stereo fluorescence microscope 24h post-inoculation. White dots in these pictures are indicative of callose deposition. Scale bars=500 μm. (B) Average number of callose deposits per 0.5mm^2^ area. Error bars represent SD values from three leaves of each plant and three independent experiments. Six microscopic fields from each leaf were analysed. * indicates (*P*<0.001) significantly different callose deposits induced by the *Xcc gumD* mutant compared with either the wild-type *Xcc*8004 strain or the Δ*rpfF*-*gumD* double mutant as determined by two-tailed Student’s *t*-test.

Xanthan has been proposed to be involved in the suppression of the plant defence response and promotion of pathogenesis presumably by suppressing host callose synthesis (Yun *et al.*, 2006). It has been reported earlier that *Xcc* mutants deficient in EPS production induced higher amounts of callose deposition in *N*. *benthamiana* leaves. A higher amount of callose deposition was also observed in *N*. *benthamiana* leaves that were infiltrated with the *gumD* mutant alone compared with those infiltrated with the wild-ype *Xcc*8004 strain (Fig. 7A, B). To investigate further the interplay between DSF and EPS (xanthan) in *Xcc*–plant interaction, a Δ*rpfF*-*gumD* double mutant was made. The callose deposition assay in *N*. *benthamiana* leaves indicated that the Δ*rpfF*-*gumD* double mutant induced significantly lower amounts of callose deposits compared with the *gumD* mutant (Fig. 7A, B). The results indicated that the higher amount of defence response-associated callose deposition induced by the EPS-deficient mutants of *Xcc* is largely due to the production of DSF. It has been shown that infiltration of 2-deoxy-d-glucose (2DDG), an inhibitor of callose synthesis (Jaffe and Leopold, 1984), could reduce defence response-associated callose deposition induced by *Xcc* strains in *N*. *benthamiana* (Yun *et al.*, 2006). To examine whether the DSF-induced callose deposition can be inhibited by 2DDG, pre-treatment or co-infiltration studies with 2DDG were carried out. The callose deposition assay indicated that either pre- or co-infiltration of 2DDG with DSF significantly reduced the callose deposition in *N*. *benthamiana* leaves compared with DSF alone (Supplementary Fig. S8 at *JXB* online).

Since *Xcc* xanthan could suppress DSF-induced callose deposition, next it was determined whether *Xcc* xanthan could rescue the defence response-associated callose deposition and growth defect exhibited by the wild-type *Pss*B728a expressing the *Xcc rpfF* [*Pss*B728a (pRpfF)]. Leaves of *N*. *benthamiana* were co-infiltrated with wild-type *Pss*B728a harbouring the *Xcc rpfF Pss*B728a (pRpfF) and the empty vector control *Pss*B728a (pHM1) with or without *Xcc* xanthan. Callose deposition and *in planta* growth assays indicated that exogenous *Xcc* xanthan suppressed the induced callose deposition as well as rescued the reduced growth phenotype exhibited by *Pss*B728a (pRpfF) (Fig. 5; Supplementary Fig. S9A–E at JXB online).

### Pre-treatment or co-inoculation with DSF inhibits the growth of *Xcc*8004 and reduces the severity of disease symptoms in *N*. *benthamiana*

**Fig. 8.**
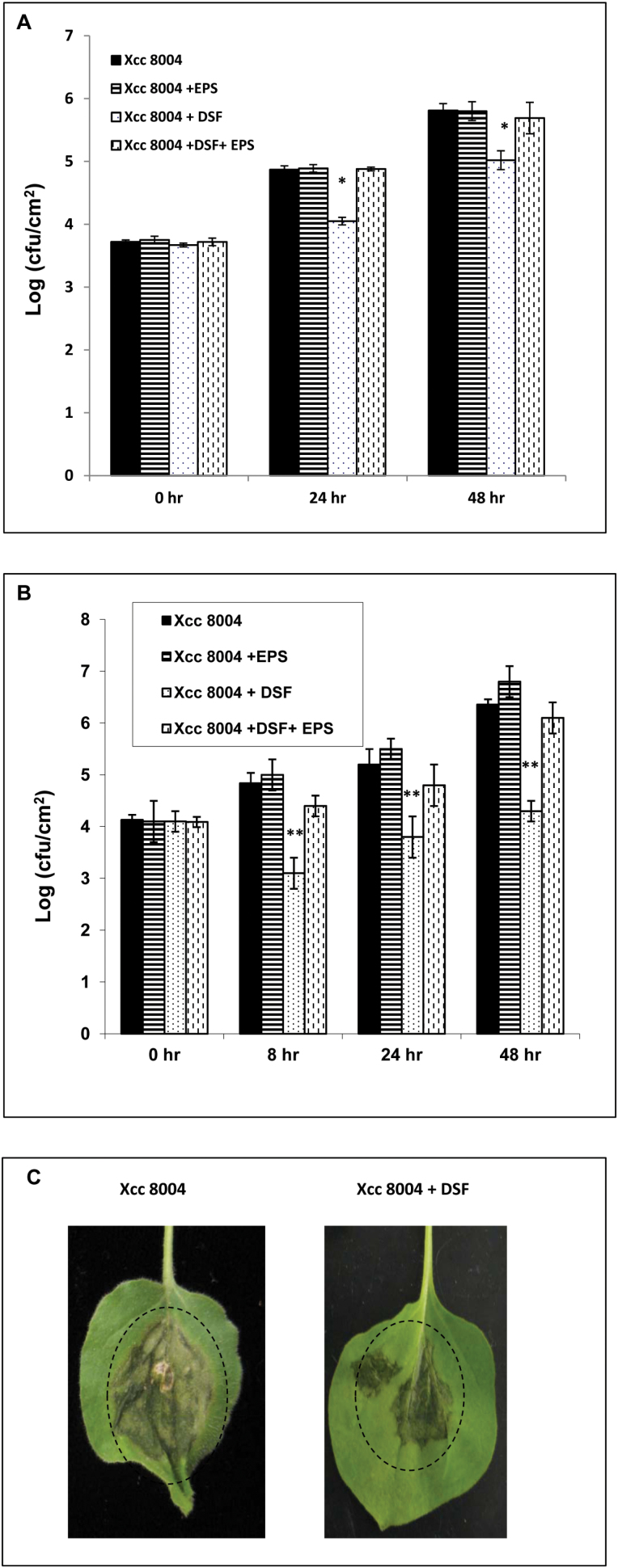
Pre-treatment or co-infiltration with DSF inhibits wild-type *Xcc*8004 growth in *N*. *benthamiana* leaves. (A) Leaves of 4-week-old plants were pre-infiltrated with either control buffer (black bars), EPS (xanthan; 0.5mg ml^–1^), DSF (100 μM), or DSF+EPS (xanthan; 0.5mg ml^–1^) by syringe infiltration 16h prior to inoculation with a 10^7^ cfu ml^–1^ suspension of the wild-type *Xcc*8004 strain. Bacterial populations were measured at 0, 24, and 48h post-inoculation. Values presented are average log (cfu cm^–2^) from six leaves (three leaves each from two independent experiments). * indicates (*P*≤0.05) a significantly lower bacterial population in the DSF-pre-inoculated leaves compared with the leaves inoculated with the wild-type *Xcc*8004 strain alone based on a pairwise Student’s *t*-test. (B) Bacterial growth assay in *N*. *benthamiana* leaves co-infiltrated with the *Xcc*8004 wild-type strain with either water control (black bars), DSF (100 μM), EPS (xanthan; 0.5mg ml^–1^), or DSF+EPS (xanthan). The bacterial population was measured at 0, 8, 24, and 48h after inoculation. Values presented are average log (cfu ml^–1^) from six leaves (three leaves each from two independent experiments). * indicates (*P*≤0.001) a significantly lower bacterial population in the DSF-co-inoculated leaves compared with the leaves inoculated with wild-type *Xcc* alone based on a pairwise Student’s *t*-test. (C) Photographs of representative leaves from the co-inoculation experiments 4 d post-inoculation. *N*. *benthamiana* leaves exhibit water soaking-like disease symptoms when inoculated with the *Xcc*8004 wild-type strain (shown in a dotted circle). Leaves co-inoculated with the *Xcc*8004 wild-type strain+DSF exhibited less vigorous water soaking symptoms compared with leaves treated with the *Xcc*8004 strain alone.

It has been demonstrated that either pre-treatment or co-inoculation with microbial elicitors (PAMPs or MAMPs) induces disease resistance, which restricts the growth of pathogenic bacteria inoculated subsequently (Jha *et al.*, 2007; Nguyen *et al.*, 2010). To test the proposed role of DSF in inducing the defence response, *N*. *benthamiana* leaves were pre-infiltrated with 100 μM DSF 16h before inoculation with 1×10^6^ cfu ml^–1^ suspensions of the wild-type *Xcc*8004 strain. Bacterial growth in the zone of infiltration was measured at 0, 24, and 48h post-inoculation (Fig. 8A). The number of wild-type *Xcc*8004 bacteria recovered from leaves pre-treated with DSF was significantly less (~7-fold) than the number recovered from leaves either pre-treated with water (control) or pre-infiltrated with DSF+xanthan (Fig. 8A).

Further, to investigate whether co-infiltration of DSF with the wild-type *Xcc*8004 strain could reduce their growth, *N*. *benthamiana* leaves were co-infiltrated with 100 μM DSF and a 10^6^ cfu ml^–1^ suspension of the wild-type *Xcc*8004 strain. Bacterial growth in the zone of infiltration was measured at 0, 8, 24, and 48h after inoculation. The number of bacteria recovered from leaves that were co-infiltrated with DSF was significantly less (~15- to 20- fold) as compared with those either infiltrated with water (control) or infiltrated together with DSF and xanthan (Fig. 8B). Interestingly, *N*. *benthamiana* leaves co-infiltrated with wild-type *Xcc*8004 and DSF exhibited reduced disease symptoms (water soaking) compared with leaves inoculated with the wild-type *Xcc*8004 strain alone (Fig. 8C).

### Pre-treatment with DSF induces resistance to *Xanthomonas oryzae* pv. *oryzae* in rice

*Xoo*, a member of the *Xanthomonas* group of phytopathogens, causes serious disease of rice known as bacterial leaf blight (Niňo-Liu *et al.*, 2006). It has been demonstrated that in *Xoo*, DSF is required for co-ordination of virulence-associated functions (Chatterjee and Sonti, 2002; Rai *et al.*, 2012). Since DSF mediated cell–cell signalling is conserved among many *Xanthomonas*, experiments were carried out to determine whether the defence response provoked by DSF would provide resistance against subsequent *Xoo* infection. Previously, it was demonstrated that pre-treatment of rice leaves with Type II effectors such as cellulase and lipase, which function as potential DAMPs, provides resistance against subsequent *Xoo* infection (Jha *et al.*, 2007; Aparna *et al.*, 2009). The midveinal regions of rice leaves were injected with DSF, buffer, and cellulase. Approximately 24h later, the leaves were inoculated with the 1×10^9^ cfu ml^–1^ suspension of the wild-type *Xoo* (BXO43) strain, 1–2cm above the point of pre-treatment. The wild-type *Xoo* strain was able to cause lesions, as shown by browning of the midvein and adjacent regions, in the majority of the leaves (80–100%) that had been pre-injected with buffer (Table 1; Supplementary Fig. S10 at *JXB* online). However, the wild-type *Xoo* strain was either unable to cause lesions or exhibited lesions of reduced lengths in leaves pre-injected with either DSF or cellulase (Table 1; Supplementary Fig. S10).

**Table 1.** *Pre-treatment of rice leaves with DSF induces resistance to subsequent* Xanthomonas oryzae *pv.* oryzae *infection*

Treatments^*a*^	Efficiency of infection (%)^*b*^			Lesion length (cm)^*c*^		
	Exp 1	Exp 2	Exp 3	Exp 1	Exp 2	Exp 3
Buffer	80 (8/10)	83 (10/12)	100 (20/20)	7.2±3.7	10.2±6.5	10.05±4.3
DSF (100 μM)	50 (5/10)	66.6 (8/12)	70 (14/20)	1.2±1.9*	2.7±2.6*	2.7±2.8*
Cellulase (1mg ml^–1^)	30 (3/10)	50 (6/12)	64.2 (9/14)	1.4±1.1*	3.3±4.6*	4.0±4.3*

^*a*^ The midvein of rice leaves of 40-day-old susceptible rice plants (TN-1) were pre-injected either with buffer alone or with buffer containing 100 μM DSF or cellulase (1mg ml^–1^). After 24h, *Xanthomonas oryzae* pv. *oryzae* (1×10^9^ cfu ml^–1^) was inoculated onto the midvein, 1–2cm above the point of initial injection, by prick inoculation. Lesion lengths were measured 12 d post-inoculation.

^*b*^ The percentage efficiency of infection is presented. Values in parentheses indicate the number of leaves that exhibited infection symptoms compared with the total number of infected leaves.

^*c*^ Lesion lengths (cm) measured 12 d after inoculation. The lesion length was measured from at least 10 infected leaves from three independent experiments, and the mean values were determined ±SD. A Student’s two-tailed *t*-test for independent mean was performed in pairwise combinations for each treatment with the control (buffer). *P<0.005 indicates a significant difference in comparison with the buffer treatment.

## Discussion

In this study, it was demonstrated that DSF (*cis*-​11-​methyl-​2-​dodecenoic acid), a widely conserved cell–cell signalling molecule from the *Xanthomonas* group of phytopathogens, induces basal defence response in *N*. *benthamiana*, *Arabidopsis*, and rice. DSF induces a HR-like response, PCD, the accumulation of autofluorescent compounds, including callose deposition, accumulation of H_2_O_2_, and expression of the *PR-1* gene. Both direct and pre-treatment with DSF induce callose deposition as well as disease resistance against subsequent infection by wild-type *Xcc* and *Xoo*, respectively. The results clearly indicated that the infiltration of higher concentrations of DSF (0.2–1mM) results in visible HR-like symptoms. However, at lower concentrations (in the range of 20–100 μM), DSF induces callose deposition without any visible HR-like symptoms (Fig. 2). It is also possible that a certain percentage of DSF infiltrated in the leaf is degraded or metabolized by plant factors, which could be one of the reasons for the fact that a higher concentration of DSF is required to elicit a defence respose compared with other strong elicitors such as bacterial flagellin. However, the *in planta* DSF production assay using the wild-type *Xcc* and *rpfF* mutant (8523) strain infiltrated with standard DSF indicated that the *Xcc* wild-type strain produces a considerably high amount of DSF at 24–48h post-inoculation (~40–100 μM) (Supplementary Fig. S5 at *JXB* online).

In a recent study, it was demonstrated that plants pre-treated with a lower concentration of AHL (a QS signalling molecule) exhibited an induced defence response (MTI) on subsequent challenge with flg22 (Schenk *et al.*, 2014). It has been proposed that AHL primes plants for the cell wall-mediated defence response that may be involved in inducing resistance to subsequent bacterial infection. The present results indicate that a low DSF concentration (10 μM) does not induce any visible defence response (callose deposition). However, leaves pre-infiltrated with 10 μM DSF exhibit induced callose deposition on subsequent challenge with flg22 (a potential bacterial MAMP) (Supplementary Fig. S4 at *JXB* online). It is possible that at an early stage of infection, when the DSF levels are low, it primes plants for cell wall-based defence response and may influence MTI or ETI (elicitor-triggered immunity).

An increasing body of research now indicates that both exogenous and endogenous unsaturated fatty acids play an important role in the plant defence response and can influence plant–microbe interactions (Upchurch, 2008; Kachroo *et al.*, 2001, 2003; Savchenko *et al.*, 2010). Exogenous fatty acids not commonly abundant in plants, such as eicosapentaenoic acid and arachidonic acid, act as potent elicitors of the defence response in solanaceous plants (Bostock *et al.*, 1981; Knight *et al.*, 2001). Arachidonic acid, a *cis*-unsaturated fatty acid characteristic of oomycete pathogens belonging to the *Phytophthora*, acts as a potent elicitor of PCD and the defence response in plants at higher concentrations (Knight *et al.*, 2001; Upchurch 2008; Savchenko *et al.*, 2010). It has also been proposed that *cis*-unsaturation is a critical structural feature for activity of these lipophilic fungal elicitors in inducing the defence response in plants (Bostock *et al.*, 1981, 1992; Bostock, 2005). It is pertinent to note in this regard that the DSF family of signalling molecules has a characteristic *cis*-unsaturated double bond at the 2-position and has been shown to be a key structural feature for its activity as a QS signalling molecule in the *Xanthomonas* group of phytopathogens (Wang *et al.*, 2004; Deng *et al.*, 2011). The present results indicated that the ability of DSF to induce the plant defence response is specific, as infiltration of a range of fatty acid compounds including *trans*-11-methyl dodecenoic acid did not result in visible HR-like symptoms or callose deposition (Supplementary Table S2 at *JXB* online). It is possible that similar to the DSF-sensing machinery in *Xanthomonas* phytopathogens, plants might have also evolved a machinery which could recognize the DSF family of signalling molecules containing the characteristic structural feature of a *cis*-unsaturated double bond.

The present results suggest that the production of DSF in *Xcc* bacteria is associated with the induced defence response, as the DSF-deficient *rpfF* mutant of *Xcc* (*Xcc*8523) induced reduced amounts of callose deposits in *N*. *benthamiana* leaves compared with those infiltrated with either the wild-type *Xcc*8004 strain or the DSF-deficient mutant strain harbouring the wild-type *rpfF* allele (Fig. 4C, D). It is possible that the reduced callose deposition induced by the DSF-deficient *rpfF* mutant may be due to the fact that DSF may positively regulate the production of factors which can induce callose deposition, such as extracellular cell-wall-hydrolysing enzymes. Interestingly, production of DSF in a non-DSF-producing phytopathogen, *Pss*B728a, a pathogen of bean and the model plant *N*. *benthamiana* (Loper and Lindow, 1987; Vinatzer *et al.*, 2006), leads to induced callose deposition and reduction of *Pss*B728a growth in *N*. *benthamiana* leaves (Figs 4, 5). The DSF synthase RpfF has been reported to be a promiscuous enzyme which can produce closely related members of the DSF family of signalling molecules (Beaulieu *et al.*, 2013). It is also possible that B728a harbouring the *Xcc* RpfF may produce an altered DSF family of signalling molecules.

It is becoming increasingly evident that during infection, pathogens actively suppress the plant’s PAMP-triggered defence responses. Gram-negative phytopathogens secrete several effector proteins via the type III secretion system (TTSS) which are involved in the suppression of the elicitor-triggered plant defence response (Hauck *et al.*, 2003; DebRoy *et al.*, 2004; Jamir *et al.*, 2004). Apart from effector proteins, several non-proteinaceous effectors from pathogenic bacteria have been reported to play a role in suppression of the plant defence response, such as EPS, glucan, and coronatin (for reviews, see Chisholm *et al.*, 2006; Boller and Felix, 2009). The EPS xanthan is required for virulence and colonization in several members of the *Xanthomonas* group of phytopathogens. In *Xcc*, production of EPS is positively regulated by cell–cell signalling mediated by DSF (Barber *et al.*, 1997; Wang *et al.*, 2004). Further, it has been shown that xanthan suppresses the elicitor-induced defence response, presumably by the chelation of extracellular calcium ions and suppression of the plant defence response by inhibiting the callose biosynthesis which is involved in cell wall fortification (Yun *et al.*, 2006; Aslam *et al.*, 2008). The present results suggest that wild-type *Xcc* is able to suppress the DSF-triggered plant defence response by production of the EPS xanthan (Fig. 6). The *Xcc* mutants defective in xanthan production induced significantly higher amounts of callose deposits and were unable to suppress DSF-induced callose deposition compared with the wild-type strain (Figs 6, 7). Further, it has been demonstrated that the DSF- and EPS (xanthan)-deficient double mutant (Δ*rpfF*-*gumD*) of *Xcc* induced significantly lower amounts of callose compared with that induced by the EPS-deficient *gumD* mutant (Fig. 7). These results suggest that the defence responses that are provoked by the EPS-deficient *gum* mutants are mostly contributed due to production of DSF.

**Fig. 9.**
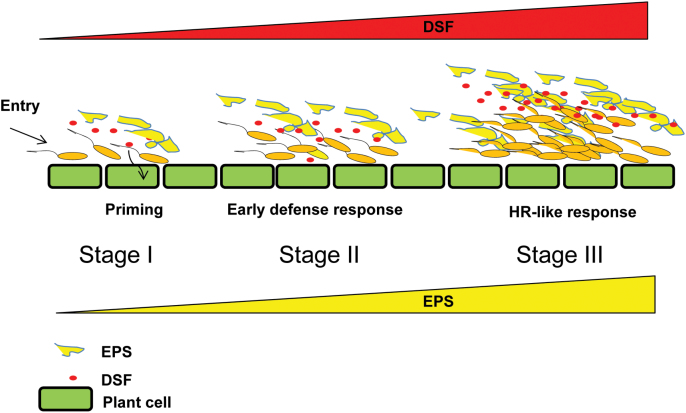
Proposed model for functional interplay between diffusible signalling factor (DSF) and extracellular polysaccharide (EPS) in *Xanthomonas*–plant interaction. At the initial stage of infection and colonization (stage I), *Xcc* gains entry through hydathodes or stomata, and colonizes in the xylem vessel. At this stage (at low cell density; stage I), the production of DSF and EPS is low. At lower concentrations of DSF (presumably ≤10 μM), DSF may be involved in priming (sensitization) plants for MTI (MAMP-triggered immunity) mediated by MAMPs such as flagellin or LPS (lipopolysaccharide). MTI is further suppressed by Type III secretion system effectors. At stage II, there is increase in *Xcc* cell number, which is associated with increased production of DSF and EPS. An increased DSF level (≥20 μM) induces an early plant defence response (callose deposition), which is suppressed by EPS. At a late stage of infection (stage III), there is a further increase in cell density due to growth of *Xcc in planta*. Due to high cell density, a high level of DSF is produced (50–100 μM). This may lead to a further increase in the production of EPS, a virulence-associated factor positively regulated by DSF. A high EPS level can suppress the plant defence response provoked by DSF including early HR-like symptoms.

In *Xcc*, DSF-mediated cell–cell signalling positively regulates the production of several secreted virulence factors such as EPS and cell-wall-degrading hydrolytic enzymes in a density-dependent manner. EPS is required for virulence and is involved in biofilm formation, a social behaviour important for colonization. A model is proposed that elucidates the functional interplay between DSF and EPS in *Xanthomonas*–plant interaction (Fig. 9). At the initial stage of infection and colonization (stage I), *Xcc* gains entry through hydathodes or stomata, and colonizes in the xylem vessel. At this stage (low cell density; stage I), the production of DSF and EPS is low. At lower concentrations of DSF (presumably <10 μM), DSF may be involved in priming (sensitization) plants for a cell wall-based defence mechanism, which may influence MTI mediated by MAMPs such as flagellin or lipopolysaccharide. MTI is further suppressed by Type III secretion system effectors. In stage II, there is increase in *Xcc* cell number, which is associated with increased production of DSF and EPS. An increased DSF level (≥20 μM) induces an early plant defence response (callose deposition), which is suppressed by EPS. At a late stage of infection (stage III), a high level of DSF is produced (50–100 μM) due to further *in planta* growth of *Xcc.* This may lead to a further increase in the production of EPS, a virulence-associated factor positively regulated by DSF. A high EPS level can suppress the plant defence response provoked by DSF including early HR-like symptoms.

Exciting further work and a challenge would be to understand the biochemical and signal transduction pathways that are involved in DSF perception and activation of plant defence responses. It will be also exciting to examine whether cell–cell signalling molecules belonging to different chemical classes from plant pathogens may also contribute to induction of host defence responses.

## Supplementary data

Supplementary data are available at *JXB* online.

Figure S1. Induction of the hypersensitive (HR)-like response by DSF isolated from the cell-free culture supernatant of the wild-type *Xcc*8004 strain by ethyl acetate extraction.

Figure S2. Response of the *Xcc* DSF biosensor strain to synthetic DSF.

Figure S3. DSF induced callose deposition in *N. benthamiana* leaves.

Figure S4. Callose deposition in *N*. *benthamiana* leaves pre-treated with DSF and subsequently challenged with flg22.

Figure S5. Detection of DSF production in *N*. *benthamiana* leaves using the *Xcc* DSF biosensor strains.

Figure S6. *In planta* growth of *Pss* harbouring the *Xcc* pRpfF.

Figure S7. EPS production assay.

Figure S8. 2-Deoxy-d-glucose (2DDG) inhibits the DSF-induced callose deposition in *N*. *benthamiana* leaves.

Figure S9. Callose deposition induced by the *P. syringae Pss*B728a wild-type strain harbouring *Xcc rpfF* is suppressed by *Xcc* xanthan.

Figure S10. Pre-treatment of rice leaves with DSF induces resistance against subsequent *Xanthomonas oryzae* pv. *oryzae* (*Xoo*) infection.

Table S1. Infiltration of synthetic DSF into the leaves of rice and *Arabidopsis thaliana* induces callose deposition.

Table S2. Specificity of DSF in inducing callose deposition in *N*. *benthamiana* leaves.

Supplementary Materials and methods

Supplementary Data
