# Supplementary material for: Xanthomonas campestris cell–cell signalling molecule DSF (diffusible signal factor) elicits innate immunity in plants and is suppressed by the exopolysaccharide xanthan
Source: J Exp Bot. 2015 Aug 5;66(21):6697–714. doi: 10.1093/jxb/erv377 (PMC4623683; doi:10.1093/jxb/erv377)
Supplement: Supplementary Data [file supp_66_21_6697__index.html]

 Xanthomonas campestris cell–cell signalling molecule DSF (diffusible signal factor) elicits innate immunity in plants and is suppressed by the exopolysaccharide xanthan — Xanthomonas campestris cell–cell signalling molecule DSF (diffusible signal factor) elicits innate immunity in plants and is suppressed by the exopolysaccharide xanthan — Supplementary Data 

# *Xanthomonas campestris* cell–cell signalling molecule DSF (diffusible signal factor) elicits innate immunity in plants and is suppressed by the exopolysaccharide xanthan

## Supplementary Data

Data files

- Supplementary Data - Supplementary Data
