# Supplementary material for: Xanthomonas campestris cell–cell signalling molecule DSF (diffusible signal factor) elicits innate immunity in plants and is suppressed by the exopolysaccharide xanthan
Source: J Exp Bot. 2015 Aug 5;66(21):6697–714. doi: 10.1093/jxb/erv377 (PMC4623683; doi:10.1093/jxb/erv377)
Supplement: Supplementary Data [file supp_erv377_edited_supp_erv377___Copy.pdf]

Supplementary data

Supplementary Tables

Table S1. Infiltration of synthetic DSF into the leaves of rice and *Arabidopsis thaliana* induces callose deposition.

DSF concentration[a]	**Average number of callose deposits per 0.5 mm^2 area ± SD[b]**	
	**Rice**	***Arabidopsis thaliana***
0 µM (Control)	6.8 ± 4.4	8.3 ± 5.0
50 µM	27.3 ± 10.1*	31.9 ± 8.1*
100 µM	42.7 ± 6.6*	60.7 ± 11.13*
200 µM	60.6 ± 10.3*	110.7 ± 25.8*

[a] DSF was infiltrated with 1ml needleless syringe on fully expanded leaves of either the adaxial surfaces of 15-day-old rice (susceptible cv. Taichung Native-1 [TN-1]) or 7-week old *Arabidopsis thaliana* ecotype Columbia (Col-0). For control treatment 1% methanol in water was used. [b]Callose deposition was visualized by staining with aniline blue and visualized by the stereo florescent microscope 18 h post infiltration. Results shown are the mean number of callose deposits per 0.5 mm^2 of leaf tissue and standard deviation from at least four leaves in each experiment (three experiments). For each leaf six microscopic fields were analyzed. * indicate P

< 0.01 (T-test) significant difference between the data obtained for the DSF treatment compared to those obtained from the control (1% methanol in water).

Table S2. Specificity of DSF in inducing callose deposition in *N. benthamiana* leaves.

	**Average number of callose deposits per 0.5 mm^2 area ± SD**[b]	
**Treatment**[a]	**Experiment 1**	**Experiment 2**
Control	18.7 ± 9.6	15 ± 6.5
DSF (100 µM)	94.4 ± 16.3*	115 ± 20.4*
Trans-11-methyl dodecenoic acid (1 mM)	27.4 ± 19.04	n. d
decanoic acid (1 mM)	22.0 ± 8.2	n. d
Lauric acid (1mM)	32.3 ± 16.3	n. d
Palmitic acid (1 mM)	23.2 ± 14.7	n. d
Myristoleic acid (1mM	n. d	38 ± 10.5
Palmitoleic acid (1 mM)	n. d	30.4 ± 12.6

[a]*N. benthamiana* leaves were infiltrated with one of the following: control (1% methanol in water), DSF, *trans*-11-methyl dodecenoic acid, decanoic acid, lauric acid, palmitic acid, myristoleic acid and palmitoleic acid.

[b]Callose deposition was visualized by staining with aniline blue and visualized by the stereo fluorescence microscope 24 h post infiltration. Results shown are the mean number of callose deposits per 0.5 mm^2 of leaf tissue and standard deviation from at least three leaves in each experiment (three experiments). For each leaf six microscopic fields were analyzed. * indicate $P < 0.01$ (T-test) significant difference between the data obtained for the DSF treatment compared to those obtained from the control and treatment with other fatty acid.

Supplementary Figures

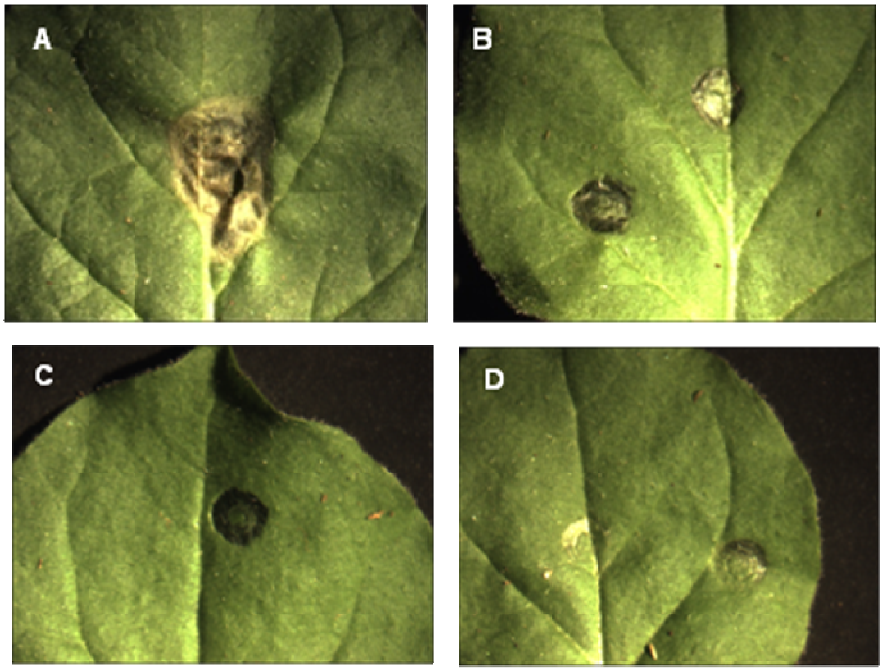

Fig. S1. Induction of hypersensitive (HR)-like response by DSF isolated from the cell free culture supernatant of wild type *Xcc*8004 strain by ethyl acetate extraction. (A to D) *N. benthamiana* leaves after infiltration with DSF isolated from wild type *Xcc*8004 strain (A); Xcc *rpfF* (DSF^-) (B); control media extract (C); and coinfiltration of DSF with Xcc8004 (D).

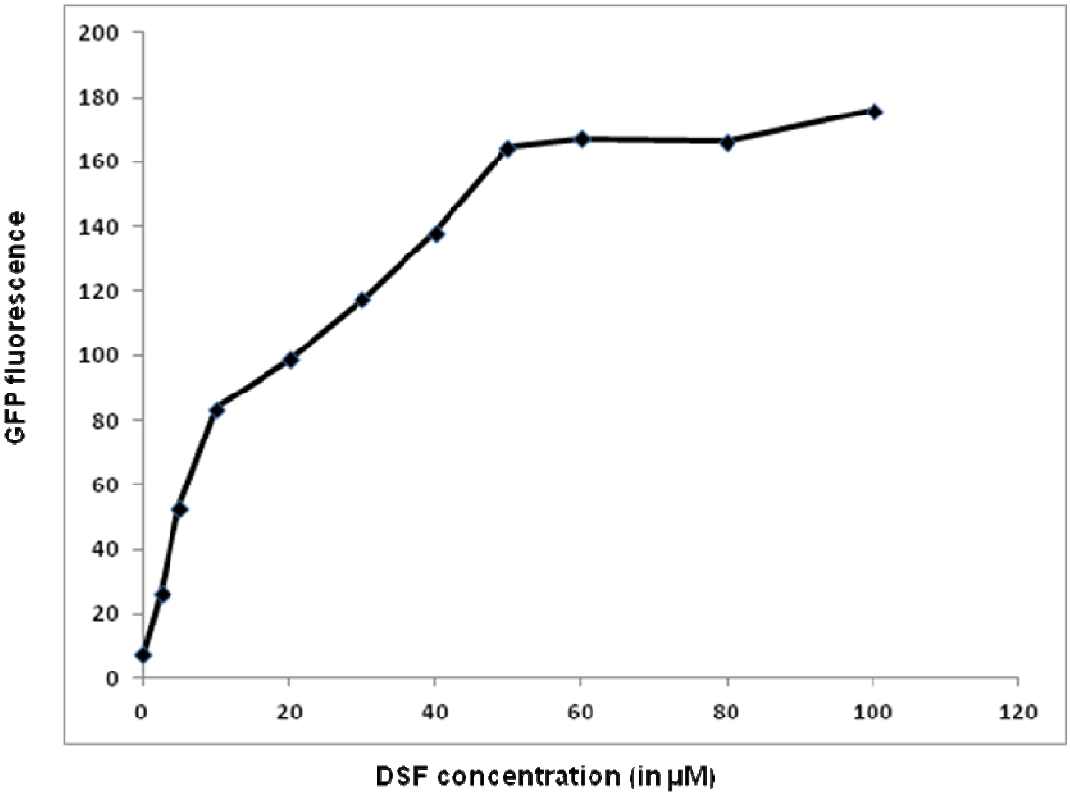

Fig. S2. Response of the *Xcc* DSF biosensor strain to synthetic DSF. *Xcc* 8523 (pKLN55) strain harbouring a transcriptional fusion of GFP with the endoglucanase (*eng*) promoter (*P eng: gfp*) was grown in the presence of different concentration of synthetic DSF for 30 h. Y-axis indicates average whole cell GFP fluorescence determined by fluorescence spectrophotometer (GFP fluorescence intensity at Excitation.-472 nm, Emission.-512 nm).

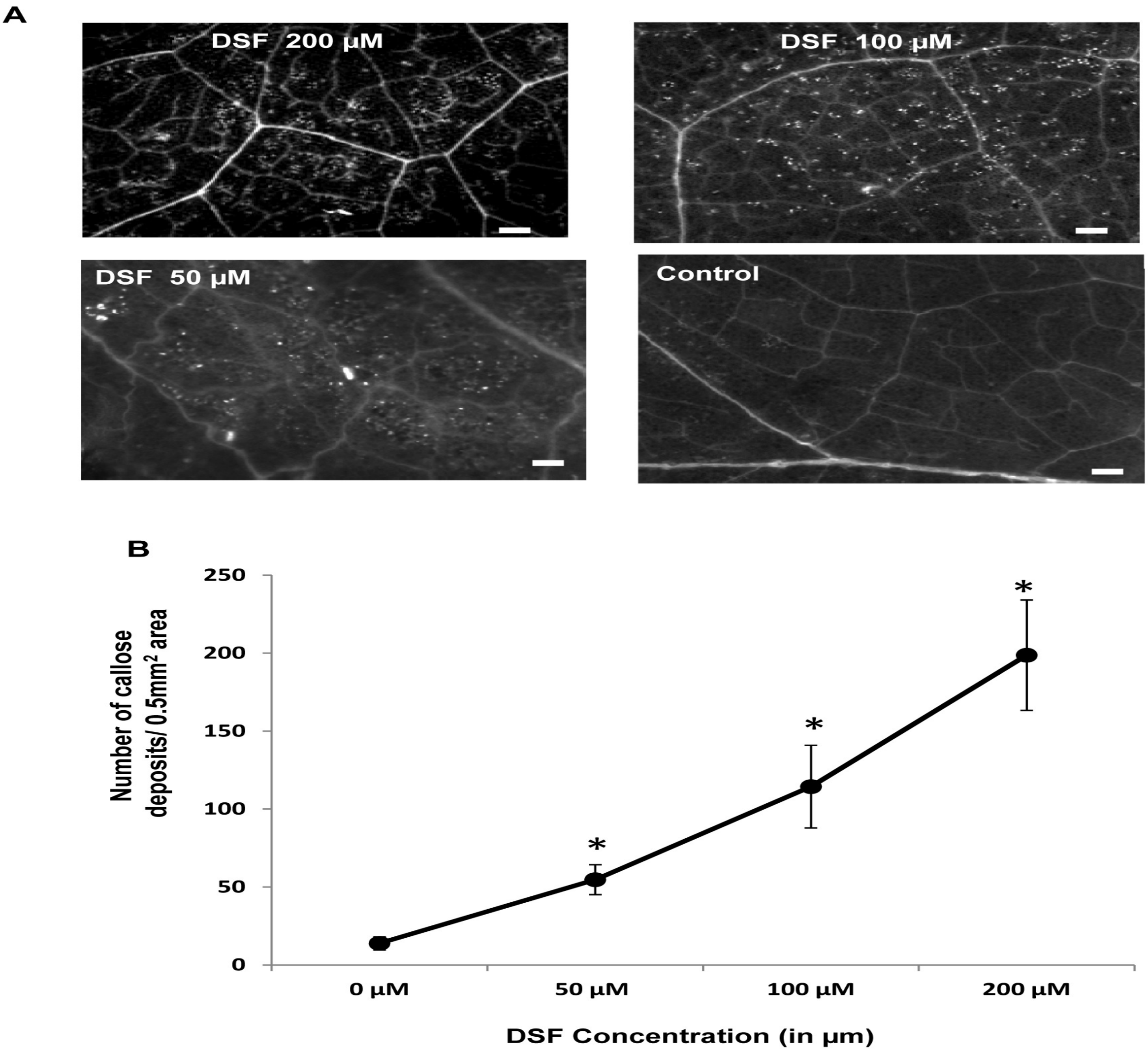

Fig. S3. DSF induced callose deposition in *N. benthamiana* leaves.

(A) *N. benthamiana* leaves were infiltrated with (Left to right) 200, 100, 50 µM DSF and control (0 µM), visualized for callose deposition 18 h post infiltration. Bars = 500 µm. (B) Average numbers of callose deposits per 0.5 mm^2. Error bars represent SD values from four leaves from each plant in three independent experiments. For each leaf, six microscopic fields were analyzed. * indicate $P < 0.01$, significant differences between the responses to the DSF treatment compared to the control (indicated by 0 µM) as assessed by Student's t test.

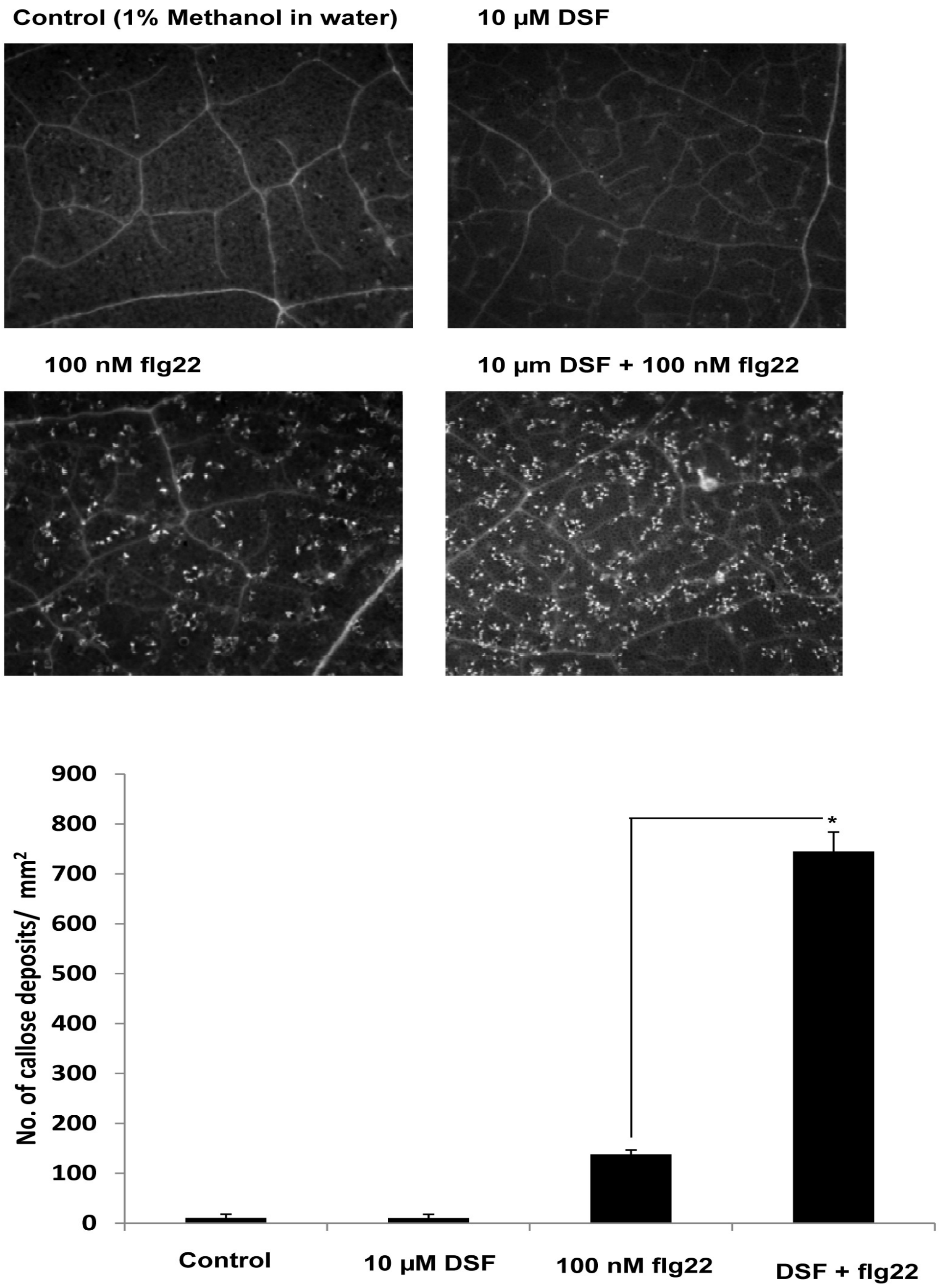

Fig. S4. Callose deposition in *N. benthamiana* leaves pretreated with DSF and subsequently challenged with flg22.

(A) *N. benthamiana* leaves were pre-infiltrated with 10 µM DSF or 1% methanol (solvent control) for 16 h prior to challenge with 100 nM flg22 for 18 h and stained with aniline blue to

visualize callose deposition by epifluorescence microscope. Bars = 500 µm. (**B**) Average numbers of callose deposits per mm^2. Error bars represent SD values from four leaves from each plant in three independent experiments. For each leaf, six microscopic fields were analyzed. * indicates $p<0.01$ in student's *t* test.

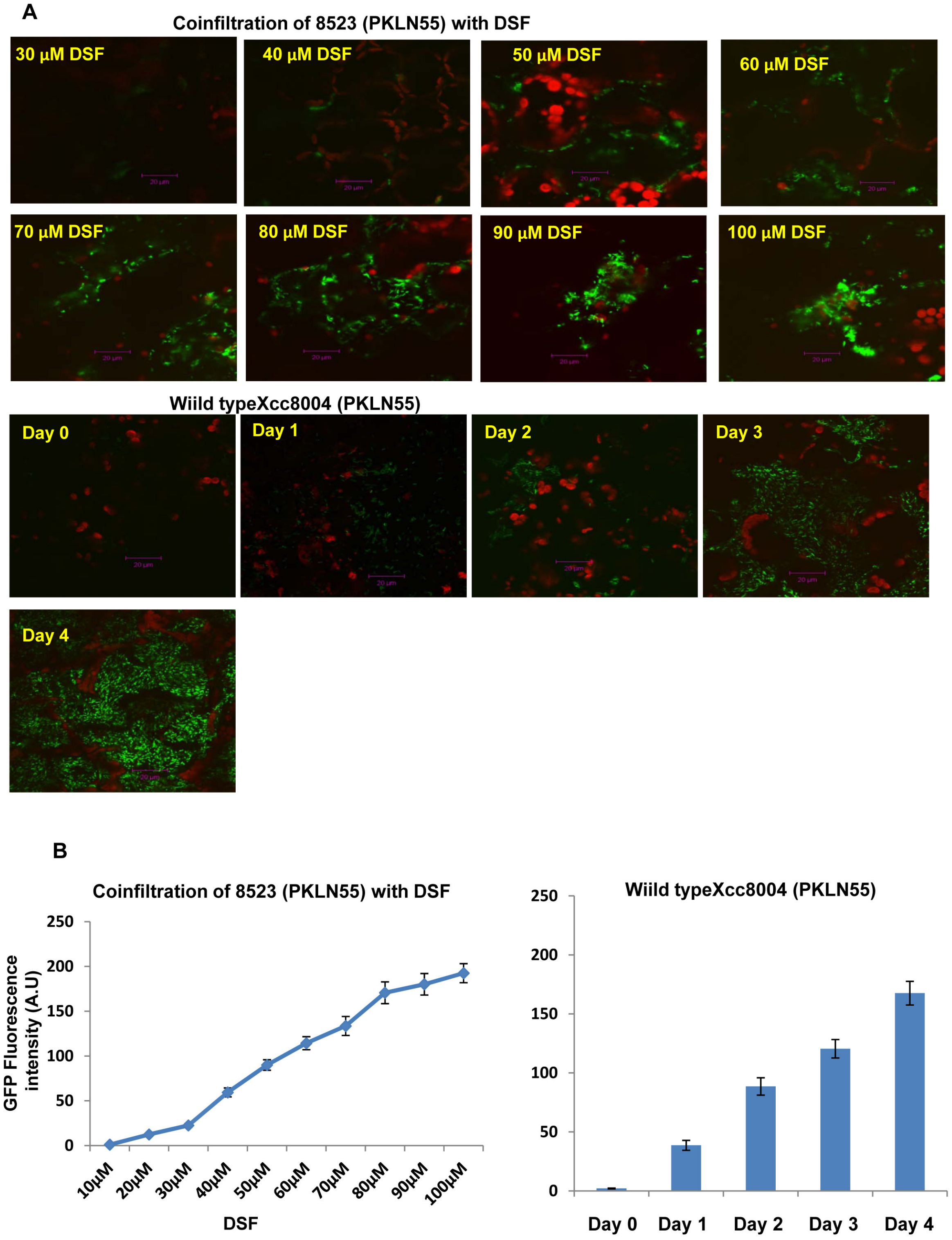

Fig. S5. Detection of DSF production in *N. benthamiana* leaves using the Xcc DSF biosensor strains.

The wild type Xcc8004 (pKLN55) and 8523 (pKLN55) strains were grown overnight for 12 h to a density of 1 X 10^6 C.F.U/ml, similar to cultures used in the infiltration experiments. At low cell density (1 x10^6 C.F.U /ml), the Xcc8004 DSF biosensor strain exhibits low GFP fluorescence, which is indicative of low DSF production in PS media (Pradhan and Chatterjee 2014). For estimating DSF levels *in planta*, *N. benthamiana* leaves were infiltrated with either wild type Xcc8004 (pKLN55) or 8523 (pKLN55) coinfiltrated with different concentrations of DSF. Leaves were analyzed by Confocal Laser Scanning Microscopy (CLSM). (A) Representative CLSM of leaves infiltrated with 8523 (pKLN55) +DSF or 8004 (pKLN55). Scale bar: 20 µm. Excitation maximum was at 488 nm (argon laser) and emissions were collected at 510 to 530 nm (for EGFP fluorescence) and 650 to 710 nm (for leaf red auto fluorescence). The panels depict confocal microscope based projection images (130 by 130 by 32 μm^3 in the X, Y and Z axis beginning from the dorsal surface) of *N. benthamiana* leaves. (B) The mean GFP pixel intensity of ~50 bacterial cells of 8523 (pKLN55) were measured and compared with the mean GFP fluorescence intensity of wild type Xcc8004 (pKLN55) after 0, 1, 2, 3 and 4-day post inoculation. Approximately, 50 cells per field were observed and 10-15 fields were counted per leaf (six leaf each from three independent experiments were analyzed). Error bars represent SEM.

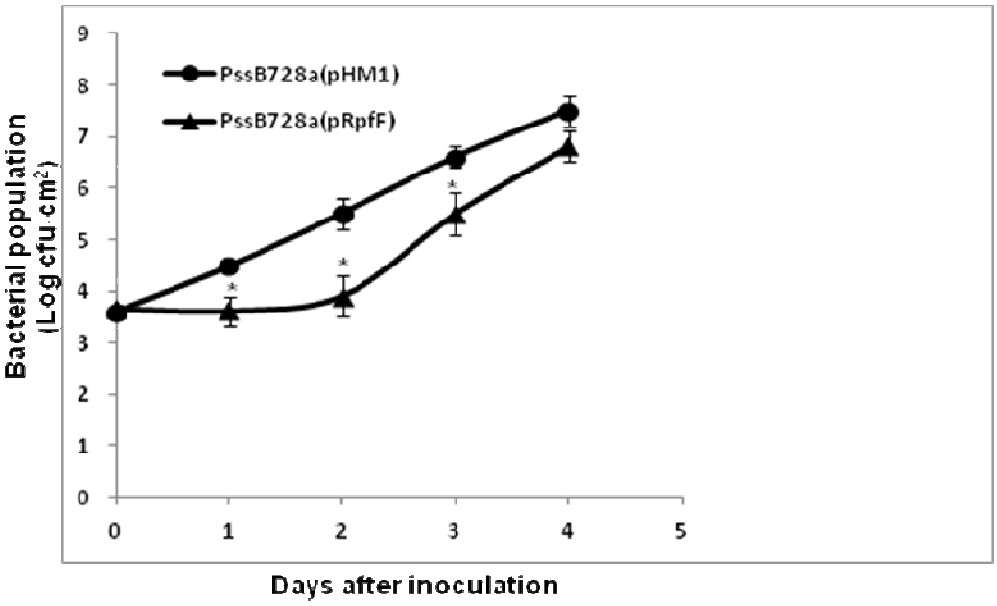

Fig. S6. *In planta* growth of *Pss* harboring the Xcc pRpfF.

Leaves of 4-week -old *N. benthamiana* were infiltrated with wild type *Ps*s harboring the plasmid containing the DSF synthase (pRpfF) or the empty vector (pHM1). Bacterial population was measured at 0, 1, 2, 3 and 4 days post inoculation from six 1 cm^2 leaf disc areas around the infiltration zone. Values presented are average log (cfu/cm^2) from six leaves each from two independent experiments. * indicate $p \leq 0.05$, significantly different population of bacteria compared with the wild type *Pss* harboring the empty vector (pHM1) control based on a pair wise Student *t* test.

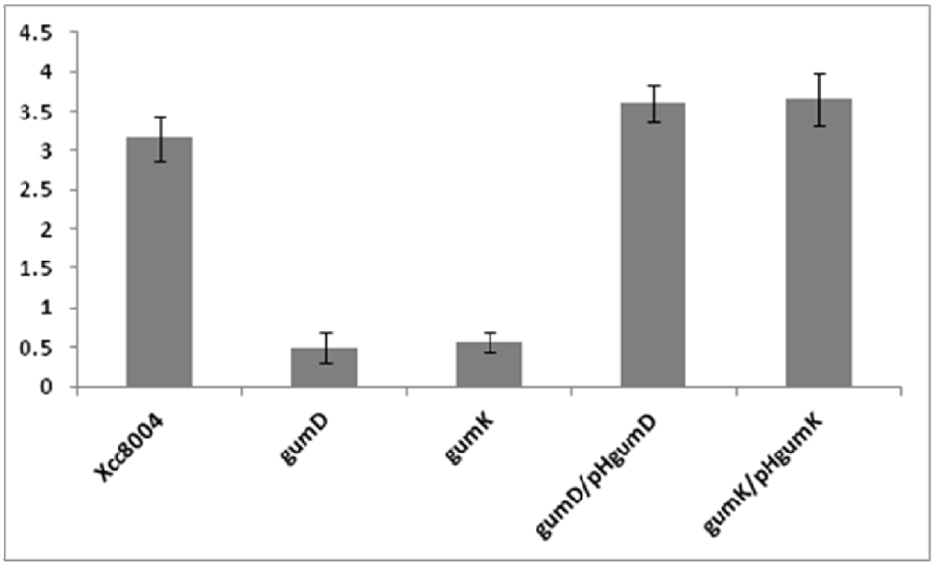

Fig. S7. EPS production assay. Different strains of *Xcc* were grown in PS medium overnight and EPS was isolated by acetone precipitation and quantitated by phenol sulphuric acid method. Xcc8004 (wild type), *gumD, gumK, gumD/*pHgumD (*gumD* mutant harboring the wild type *gumD* gene in complementing plasmid) and *gumK*/pHgumK(*gumK* mutant harbouring the wild type *gumK* gene in complementing plasmid) strains were used. Error bars represents average EPS production from three independent experiments each with three replicates.

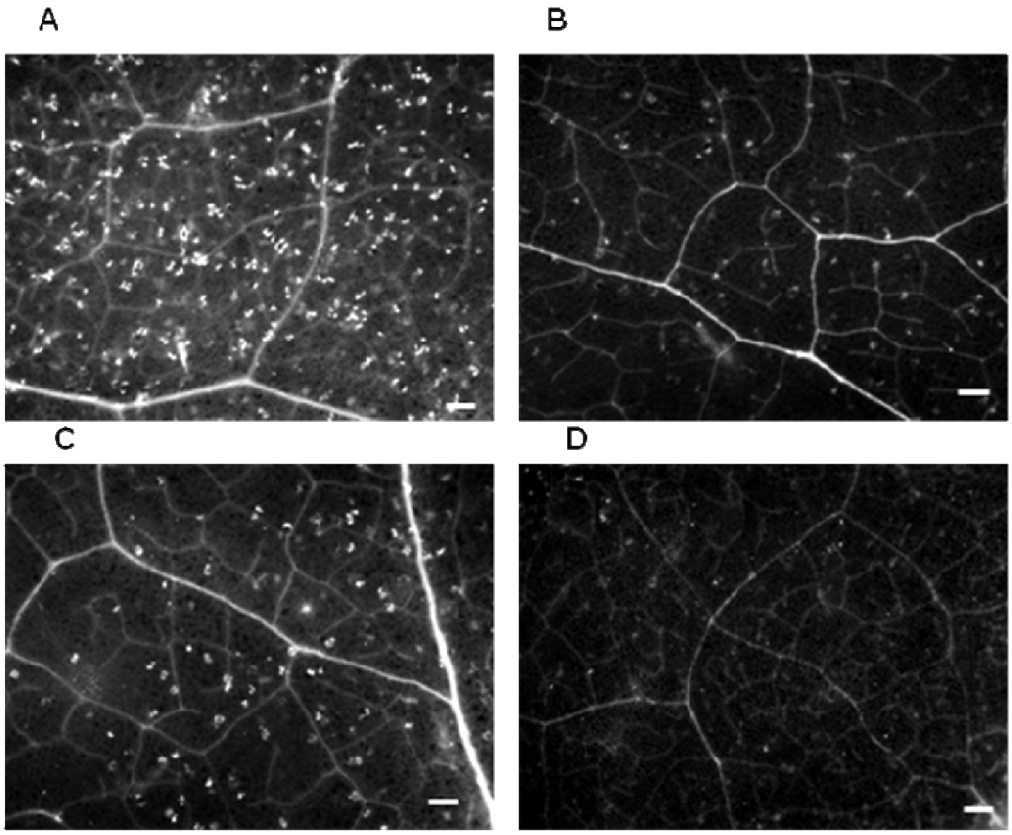

Fig. S8. 2-deoxy-D-Glc (2 DDG) inhibits the DSF induced callose deposition in *N. benthamiana* leaves. *N. benthamiana* leaves were pretreated with either (A) control (1% methanol in water) or (B) 250 µM of 2 DDG and subsequently after 8 h, infiltrated with 1mM DSF. (C) Coinfiltration of 250 µM DDG with 1 mM DSF suppressed DSF induced callose deposits. (D) Water control. Callose deposition was visualized by staining with aniline blue and visualized by sterio fluorescence microscope. White dots in these pictures are indicative of callose deposition. Bars = 500 µm.

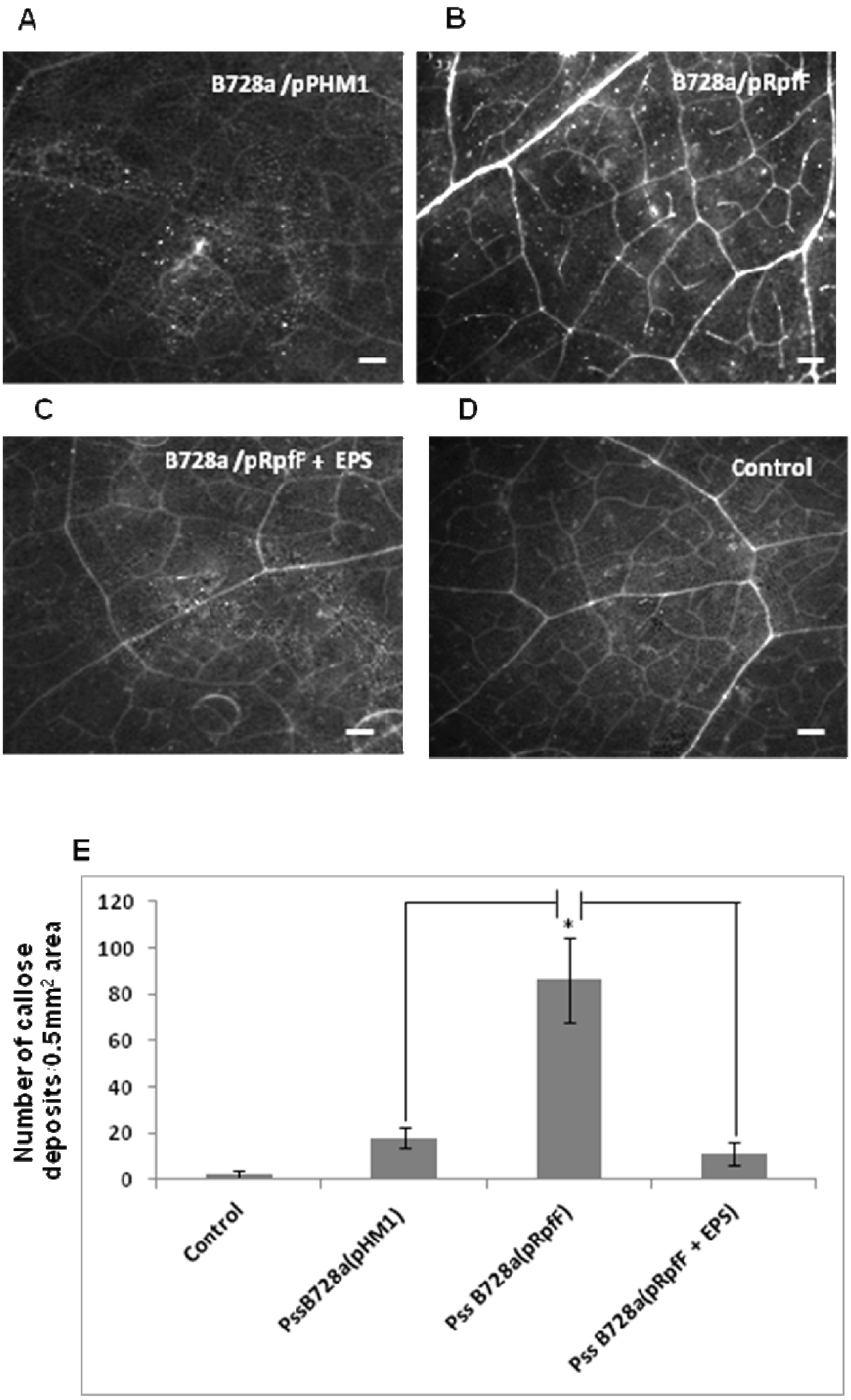

Fig. S9. Callose deposition induced by the *P. syringae Pss*B728a wild type strain harboring the Xcc *rpfF* is suppressed by *Xcc* xanthan. *N. benthamiana* leaves infiltrated with 1 x 10^6 C.F.U/ ml

suspension of different *Pss* strains; (A) B728a (pHM1; empty vector control); (B) B728a (pRpfF); (C) coinfiltration of B728a (pRpfF) with *Xcc* xanthan (0.5 mg/ml); and (D) control (water). Callose deposition was visualized by staining with aniline blue and visualized by fluorescence microscope 24 h post inoculation. Bars = 500 µm. (E) Average number of callose deposits per 0.5 mm^2 area. Error bars represent SD values from four leaves of each plant in three independent experiments. Six microscopic fields from each leaf were analyzed. * Indicates ($p < 0.001$) significantly different callose deposits induced by the *Pss*B728a (pRpfF) compared with either the B728a (pHM1) strain or coinfiltration of *Pss*B728a (pRpfF) strain with *Xcc* xanthan as determined by two tail Student't' test.

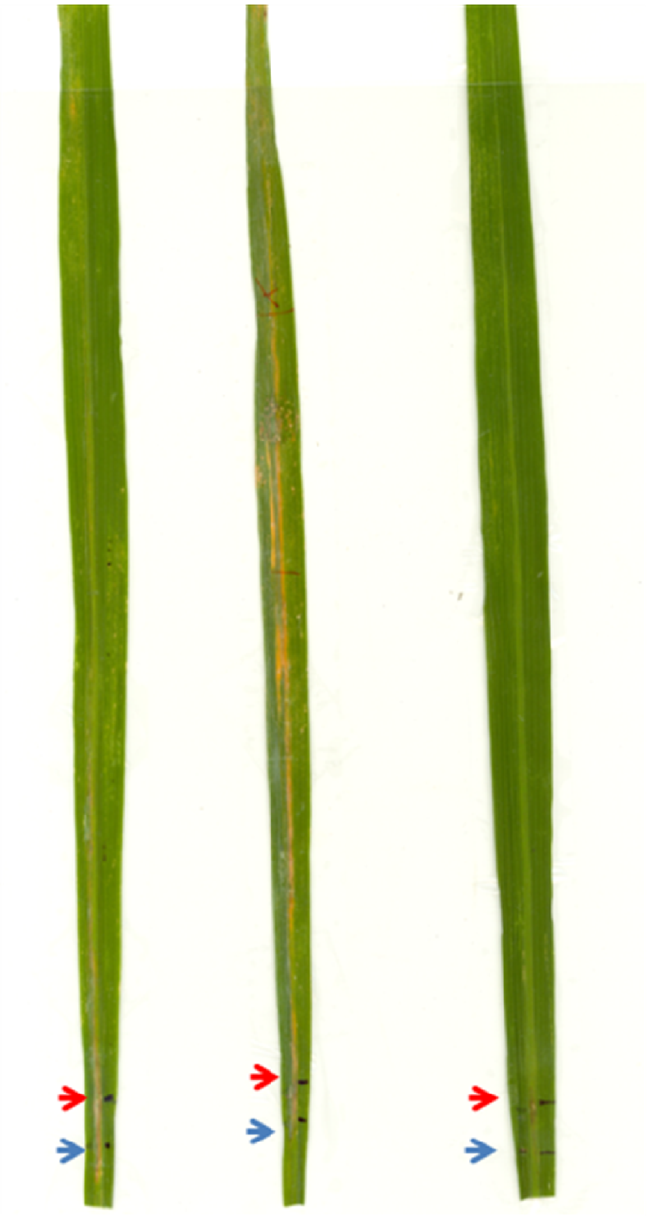

Fig. S10. Pretreatment of rice leaves with DSF induces resistance against subsequent *Xanthomonas oryzae* pv. *oryzae* (*Xoo*) infection. The midveins of rice leaves were preinjected with buffer (10mM KH_2P0_4), DSF (100 µM) and cellulase (0.1 mg/ml) from *Aspergillus niger*. After 24 h, the Xoo wild type strain was inoculated 1 to 2 cm above the point of pretreatment. Disease lesion (discoloration of midveinal regions formed in the leaves were scored 12 days post inoculation of *Xoo*. Disease lesion (discoloration of midveinal regions) formed on leaves

pretreated with buffer, but were either absent or very much reduced in leaves pretreated with DSF or cellulase. Blue arrows indicate the point of pretreatment whereas red arrows indicate the point of subsequent inoculation with wild type *Xoo* strain.
